# Fatty acid synthase as a new therapeutic target for HER2-positive gastric cancer

**DOI:** 10.1007/s13402-023-00769-x

**Published:** 2023-02-08

**Authors:** Lorenzo Castagnoli, Simona Corso, Alma Franceschini, Alessandra Raimondi, Sara Erika Bellomo, Matteo Dugo, Federica Morano, Michele Prisciandaro, Silvia Brich, Antonino Belfiore, Andrea Vingiani, Maria Di Bartolomeo, Giancarlo Pruneri, Elda Tagliabue, Silvia Giordano, Filippo Pietrantonio, Serenella M. Pupa

**Affiliations:** 1 Molecular Targeting Unit, Department of Research, Fondazione IRCCS Istituto Nazionale Dei Tumori, Milan, Italy; 2 Department of Oncology, University of Torino, Candiolo, Turin, Italy; 3 Candiolo Cancer Institute, FPO-IRCCS, Candiolo, Turin, Italy; 4 Department of Medical Oncology, Fondazione IRCCS Istituto Nazionale Dei Tumori, Via Venezian 1, 20133 Milan, Italy; 5 Department of Medical Oncology-Breast Cancer Unit Clinical Translational and Immunotherapy Research, IRCCS Ospedale San Raffaele, Milan, Italy; 6 Department of Pathology and Laboratory Medicine, Fondazione IRCCS Istituto Nazionale Dei Tumori, Milan, Italy; 7 Department of Oncology and Hemato-Oncology, University of Milan, Milan, Italy

**Keywords:** Gastric cancer, HER2, Gastrospheres, FASN, Therapy resistance

## Abstract

**Purpose:**

Trastuzumab is an HER2-specific agent approved as the gold-standard therapy for advanced HER2-positive (HER2+) gastric cancer (GC), but the high rate and rapid appearance of resistance limit its clinical efficacy, resulting in the need to identify new vulnerabilities. Defining the drivers influencing HER2+ cancer stem cell (CSC) maintenance/survival could represent a clinically useful strategy to counteract tumor growth and therapy resistance. Accumulating evidence show that targeting crucial metabolic hubs, as the fatty acid synthase (FASN), may be clinically relevant.

**Methods:**

FASN protein and transcript expression were examined by WB and FACS and by qRT-PCR and GEP analyses, respectively, in trastuzumab-sensitive and trastuzumab-resistant HER2+ GC cell lines cultured in adherent (2D) or gastrosphere promoting (3D) conditions. Molecular data were analyzed in silico in public HER2+ GC datasets. The effectiveness of the FASN inhibitor TVB3166 to overcome anti-HER2 therapy resistance was tested in vitro in gastrospheres forming efficiency bioassays and in vivo in mice bearing trastuzumab-resistant GC cells.

**Results:**

We compared the transcriptome profiles of HER2+ GC cells cultured in 2D versus 3D conditions finding a significant enrichment of FASN in 3D cultures. FASN upregulation significantly correlated with high stemness score and poor prognosis in HER2+ GC cases. TVB3166 treatment significantly decreased GCSCs in all cell targets. HER2 and FASN cotargeting significantly decreased the capability to form gastrospheres versus monotherapy and reduced the in vivo growth of trastuzumab-resistant GC cells.

**Conclusion:**

Our findings indicate that cotargeting HER2 and FASN increase the benefit of anti-HER2 therapy representing a new opportunity for metabolically combating trastuzumab-resistant HER2+ GC.

**Supplementary Information:**

The online version contains supplementary material available at 10.1007/s13402-023-00769-x.

## Introduction

Gastric cancer (GC) is the third leading cause of cancer-related deaths worldwide, with unsatisfactory long-term survival following standard treatments [1]. HER2 positivity, *i.e*., HER2 immunohistochemistry (IHC) 3 + or 2 + with ISH amplification, is found in less than 20% of gastric and gastroesophageal junction cancers. HER2 testing is currently recommended by all major guidelines to select patients with metastatic GC (mGC) for first-line trastuzumab (T) treatment based on the positive results of the phase III ToGA trial [2]. However, the relatively unsatisfactory median progression-free survival (mPFS) of 6.7 months after T plus chemotherapy clearly highlights the clinical relevance of primary and acquired resistance, with a lack of long-term benefit from this targeted strategy in most mGC patients. The biological foundations of resistance to HER2 inhibition in GC rely on the high heterogeneity of HER2 expression and its complex derived proteome [3] and on the frequent coexistence of other genetic/molecular alterations [4, 5]. Therefore, the identification of new vulnerabilities to improve T efficacy is crucial to move forward from the current standard targeted treatment.

Cancer stem cells (CSCs) share several properties with normal stem cells in various oncotypes, including GC [6]. Specifically, CSCs are able to continuously self-renew and differentiate into distinct lineages, being deeply implicated in tumor initiation, invasiveness, metastases, treatment resistance and in immune escape [7, 8]. Thus, targeting molecular drivers involved in the CSC program, including the dysregulated expression/activation of HER2 and its splice variant d16HER2 in HER2+ breast [9–12] and gastric [13–15] cancers, may improve cancer patient outcomes, yielding long-lasting responses [16].

Metabolic reprogramming is a well-established hallmark of cancer [17]; in particular, lipid accumulation has been observed in many types of cancers. Increasing evidence has revealed that the rewiring of lipid metabolism plays a key role in cancer development, metastases, therapy resistance, and stemness in most malignancies [18, 19]. In this scenario, the overexpression/activity of the key anabolic multienzyme fatty acid synthase (FASN), which catalyzes the last step in the de novo biosynthesis of fatty acids (FAs), represents one of the most frequent alterations that provide cancer cells with fuel to sustain the exacerbated energetic and biosynthetic demands and signaling that are essential for their proliferation, survival and invasion [20]. In this context, several studies conducted in HER2+ breast cancer (BC) revealed the existence of a reciprocal positive feedback loop between HER2 and the lipogenic enzyme FASN [20], whose upregulation can represent an important mechanism of resistance to anti-HER2 agents [19]. In particular, the simultaneous blockade of FASN and HER2 showed marked antitumor activity in HER2+ BC models that are resistant to anti-HER2 agents [21, 22]. In contrast, the functional link between FASN and HER2 in HER2+ GC remains to be elucidated, even though it was reported that GC patients with concordant expression of FASN and HER2 are more likely to suffer a poor prognosis than patients with non concordant expression [23]. Here, we examined the expression of FASN and the functional involvement of FASN upregulation in HER2+ GC preclinical models and patients and sought to determine whether FASN inhibition is capable of overcoming anti-HER2 treatment resistance in HER2 + GC, mainly by targeting its CSCs.

## Materials and methods

### Tumor cell lines

The human HER2+ GC cell lines OE19, N87, OE33 and the human HER2-negative GC cell line MKN45 were obtained from ATCC (Rockville, MD, USA) and grown as monolayer cultures in RPMI 1640 (EuroClone, Pero, MI, Italy) with 10% FBS. GC primary cells GTR0455 were derived from PDX as described in [24] and grown as a monolayer culture in IMDM (EuroClone) with 10% FBS. All tumor cell lines were cultured at 37 °C in a humidified 5% CO_2_ atmosphere and routinely tested for mycoplasma contamination.

### Gastrosphere cultures and treatments

For sphere cultures, single dissociated OE19, N87, OE33 and GTR0455 cells were plated at a concentration of 10.000 cells/ml onto low-attachment dishes (6 wells) with an ultralow attachment surface (Corning, Corning, NY, USA). Cells were cultured in epithelial basal medium supplemented as described in [13], treated on Day 0 with trastuzumab (T) (20 μg/ml; F. Hoffmann-La Roche AG, Basel, Switzerland), pertuzumab (P) (10 μg/ml; F. Hoffmann-La Roche AG) and lapatinib (L) (10 nM; Selleck Chemical, Houston, TX, USA), and TVB3166 at appropriate dilution (Merck Life Science, Darmstadt, Germany), in monotherapy or combination, with NaCl 0.9% or DMSO as distinct diluents, and collected after 7 days. The spheres were counted microscopically on day 7, and representative images were acquired using an EVOS XL Core Cell Imaging System (Thermo Fisher Scientific, Waltham, MA, USA). The gastrosphere forming efficiency (GFE) was calculated as the ratio of the number of gastrospheres to the number of single cells that were initially seeded.

### Quantitative real-time PCR (qRT‒PCR)

Total RNA was extracted from HER2+ GC cell lines using Qiazol (Qiagen, Hilden, Germany) according to the manufacturer’s instructions. cDNA was reverse-transcribed from 1 µg of total RNA in a 20-µl volume using the High-Capacity RNA-to-cDNA Kit (Thermo Fisher Scientific) and subjected to qRT‒PCR using the Applied Biosystems *SYBR Green* dye-based PCR assay on the ABI Prism 7900HT sequence detection system (Applied Biosystems, Foster City, CA, USA). HER2 and FASN mRNA transcripts were amplified using 200 nM primers (HER2 Fw: GTGTGGACCTGGATGACAAGGG Rev: GCTCCACCAGCTCCGTTTCCTG; FASN; Fw: AACTCCAAGGACACAGTCACCAT Rev; CAGCTGCTCCACGAACTCAA). Data were normalized to GAPDH (GAPDH Fw: GCTCACTGGCATGGCCTTC Rev: CCTTCTTGATGTCATCATATTTGGC) using the comparative 2^−ΔCt^ method.

### Aldehyde-dehydrogenase (ALDH) activity assay and flow cytometry

ALDH activity was measured using an ALDEFLUOR assay kit (StemCell Technologies, Vancouver, Canada) in OE19, N87, OE33, and GTR0455 cells according to the manufacturer’s instructions. Briefly, 7.5 × 10^5^ cells were suspended in ALDEFLUOR assay buffer containing the ALDH substrate BODIPY-aminoacetaldehyde (BAAA) and incubated for 1 h at 37 °C. A specific inhibitor of ALDH, diethylaminobenzaldehyde (DEAB), was used to distinguish between ALDH-positive (ALDH +) and ALDH-negative (ALDH-) cell subsets. For double-marker immunofluorescence staining, cells were incubated for 1 h at 37 °C both with an ALDEFLUOR assay Kit (StemCell Technologies) and with PE-anti-human CD340 (erbB2/HER-2) antibody (1:100) (BioLegend, San Diego, CA, USA) to analyze HER2 expression. The cells were analyzed using a BD LSR II FACScanto cytometer (BD Biosciences, Franklin Lakes, NJ, USA). In all experiments, the data were processed using the FlowJo software package.

### Western blot analysis

Protein extracts from OE19, N87, OE33, and GTR0455 tumor cells were solubilized for 40 min at 0 °C with RIPA Lysis and Extraction Buffer (Thermo Fisher Scientific) in the presence of Halt™ Protease Inhibitor Cocktail (Thermo Fisher Scientific), mixed with gel sample buffer under reducing conditions, heated for 5 min at 95 °C, and resolved by electrophoresis on precast 4–12% bis–tris gels (Invitrogen, Thermo Fisher Scientific). Separated proteins were electrophoretically transferred onto nitrocellulose filters, stained with Red Ponceau to check protein loading, washed extensively with TBS + 0.5% Tween-20, and saturated for 1 h at room temperature in blocking solution (5% low-fat milk in TBS + 0.1% Tween-20) before the addition of primary antibodies in 3% low-fat milk in TBS + 0.1% Tween-20 for 1 h at room temperature or overnight at 4 °C with gentle shaking. The following primary mouse monoclonal antibodies were used: Ab3 c-erbB-2/HER2/neu (1:100; Calbiochem, Darmstadt, Germany); anti-HER1 (sc-03 clone) (1:1.000; Santa Cruz Biotechnology, Dallas, TX, USA); and anti-β-actin-peroxidase (AC-15 clone) (1:50.000; Sigma‒Aldrich, St. Louis, MO, USA). The following primary rabbit antibodies were used: polyclonal anti-phospho-HER2 p-*Neu* (Tyr 1248) (1:1.000; Cell Signaling Technology, Danvers, MA, USA); polyclonal anti-HER3 (sc-285 clone) (Santa Cruz Biotechnology); polyclonal anti-phospho-HER1 (Tyr 1068) (1:1.000; Cell Signaling Technologies); monoclonal anti-phospho-HER3 (Tyr 1289) (1:1.000; Cell Signaling Technologies), and monoclonal anti-FASN (1:1.000; Cell Signaling, USA). Filters were then washed extensively with TBS + 0.5% Tween-20 and incubated with HRP-conjugated goat anti-mouse Ig (1:5.000) or donkey anti-rabbit Ig (1:10.000) (Amersham GE Healthcare, Little Chalfont, UK) for 1 h at room temperature. Signals were detected using enhanced chemiluminescence (ECL, Amersham GE Healthcare). Phosphorylated and basal protein expression levels were assessed relative to those of housekeeping proteins in the same sample. Levels of phosphorylated and basal protein expression were assessed relative to those of housekeeping genes in the same sample; phosphorylated protein expression was further normalized relative to basal protein expression by Quantity One 4.6.6 (Bio-Rad, Hercules, CA).

### Patients and immunohistochemistry (IHC) staining and FASN score

Using a prospective dataset established at Fondazione IRCCS Istituto Nazionale dei Tumori (AMNESIA study, approved by IRB, INT 205/17), we retrieved patients with acquired resistance to trastuzumab and available pre- and post-treatment tissue samples. HER2 positivity was confirmed in all post-progression samples.

Tumor samples were collected at the Department of Pathology at Fondazione IRCCS Istituto Nazionale dei Tumori, Milan. Neoplastic samples were reviewed by an expert pathologist and classified according to the histopathological criteria of the World Health Organization. The immunohistochemical analysis was performed on formalin-fixed paraffin-embedded (FFPE) 2 μm tumor sections, subjected to antigen retrieval using EDTA buffer (pH 8) and then immunostained with a primary monoclonal antibody anti-human FASN (1:50; clone C20G5, #3180S, Cell Signaling Technology). Immunostaining was performed using a standard immunoperoxidase protocol followed by an anti-rabbit secondary antibody and diaminobenzidine chromogen reaction (Dako, Agilent Technologies, Santa Clara, CA, USA). FASN immunoreactivity in cancer cells was quantitatively and qualitatively evaluated on full face sections using the H-score, taking into account both the prevalence of positive cells and the staining intensity, defined as negative (0), weak (1^+^), moderate (2^+^), or intense (3^+^), using the following formula: H-score = (% of 1^+^ cells) + (% of 2^+^ cells × 2) + (% of 3^+^ cells × 3), with scores potentially ranging from 0 to 300. Images at 40X magnification were acquired with an Aperio ScanScope® XT (Leica Biosystems, Nussloch, Germany).

### FASN silencing

To knock down FASN, cells were transfected with 100 nM specific silencer siRNA (Thermo Fisher Scientific—107,315) or with *Silencer*™ Negative Control No. 1 siRNA (Thermo Fisher Scientific) using Lipofectamine 3000 (Thermo Fisher Scientific), harvested 48 h post-transfection, and examined for FASN expression at both the mRNA and protein levels.

### Cell Viability Assay (WST)

To perform the cell viability assay, OE19, N87, OE33 and GTR0455 cells were seeded in 96-well plates, cultured for 72 h and treated with various concentrations of L or TVB3166 for 24 h, 48 h, and 72 h. Cell viability was assessed by a water-soluble tetrazolium (WST) colorimetric assay (BioVision, Milpitas, CA, USA). Absorbance was measured at 450 nm using a microplate reader (iMARK™, Bio-Rad, Hercules, CA, USA). The values were normalized to the growth of the cells in the presence of the diluent DMSO. Cell proliferation inhibition (%) was calculated as [(untreated—treated)/(untreated)]X100 and plotted using GraphPad Prism 5.02.

### Gene expression profiling

Total RNA was extracted from HER2+ GC OE19 and N87 cells using Qiazol (Qiagen). After sample cleanup using the RNAeasy Kit (Qiagen) according to the manufacturer’s recommendations and using RNase-free DNase to remove contaminating genomic DNA, RNA quality and quantity were assessed using the 2200TapeStation system (Agilent) and Qubit 2.0 Fluorimetric Assay (Thermo Fisher Scientific), respectively. Gene expression experiments were performed according to the GeneChip WT Pico standard protocols (Affymetrix, Thermo Fisher Scientific). After reverse transcription, cDNA synthesis, amplification, and labeling, probes were hybridized on human ClariomD arrays for 16 h at 45 °C. After washing and staining using the GeneChip Fluidics Station, arrays were scanned using Affymetrix Gene Chip Scanner 3000 7G. Primary data were acquired using the Affymetrix GeneChip Command Scan Control version 4.0 (developed by Thermo Fisher Scientific).

### Bioinformatic analyses

Raw CEL files were preprocessed at the gene level using the robust multiarray average method [25] implemented in the oligo package [26] from Bioconductor. Probe sets were annotated using the clariomdhumantranscriptcluster.db package. Probe sets with no associated HUGO gene symbol were discarded. Multiple probe sets for the same gene were collapsed using the collapseRows function of the WGCNA package [27] according to the highest variance across samples. Differential expression analysis was carried out using limma [28]. P values were corrected for multiple testing according to the Benjamini‒Hochberg false discovery rate (FDR) method. Gene set enrichment analysis [29] was carried out in preranked mode using the fgsea package [30]. Hallmark gene sets from MSigDB [31], metabolic gene signatures from Peng et al. [32], and the consensus stemness ranking (CSR) signature from Shats et al. [33] were tested**.** Normalized gene expression data of GC patients from the GSE62254 dataset were retrieved from NCBI GEO. FPKM RNA-Seq data of stomach adenocarcinoma (STAD) patients were obtained from The Cancer Genome Atlas (TCGA) using the TCGABiolinks package [34]. Patients were stratified as HER2+ or HER2-negative (HER2-) according to the bimodal distribution of ERBB2 gene expression values (Fig. S2).

### PDX generation and xenotrials

Gastric PDX generation was performed as described [35]. All animal procedures adhered to the ‘Animal Research: Reporting of In Vivo Experiments’ (ARRIVE) standards and were approved by the Ethical Committee of the Candiolo Cancer Institute and by the Italian Ministry of Health. For xenograft experiments**,** PDXs were passaged and expanded for > 5 generations until a cohort of mice was produced. Five-week-old female nonobese diabetic (NOD)/severe combined immunodeficient (SCID) mice were used. Established and randomized tumors (average Volume 200 mm^3^) were treated for the indicated days with the following regimens (either single agent or combination): vehicle (saline) per os; trastuzumab 30 mg/kg, weekly, intraperitoneal; and TVB-3166 60 mg/kg, daily, per os. Tumor size was evaluated once weekly by caliper measurements, and the approximate volume of the mass was calculated using the formula 4/3π(D/2)(d/2)2, where D is the major tumor axis and d is the minor tumor axis. Statistical testing for pharmacological experiments was performed with GraphPad Prism Software 8.0 using two-way ANOVA followed by Bonferroni multiple comparisons correction. Statistical significance: ns = not significant; *p < 0,05; **p < 0,01; ***p < 0,001; ****p < 0,0001.

### Statistical analysis

Statistical analyses were performed using GraphPad Prism 5.02. Statistical significance was determined by two-tailed unpaired or paired Student’s t test. When p < 0.05, the difference between the compared groups was considered to be statistically significant. Data are presented as the mean ± SEM (n ≥ 3).

## Results

### Activated HER2 is a biomarker of HER2+ GCSCs

**Fig. 1:**
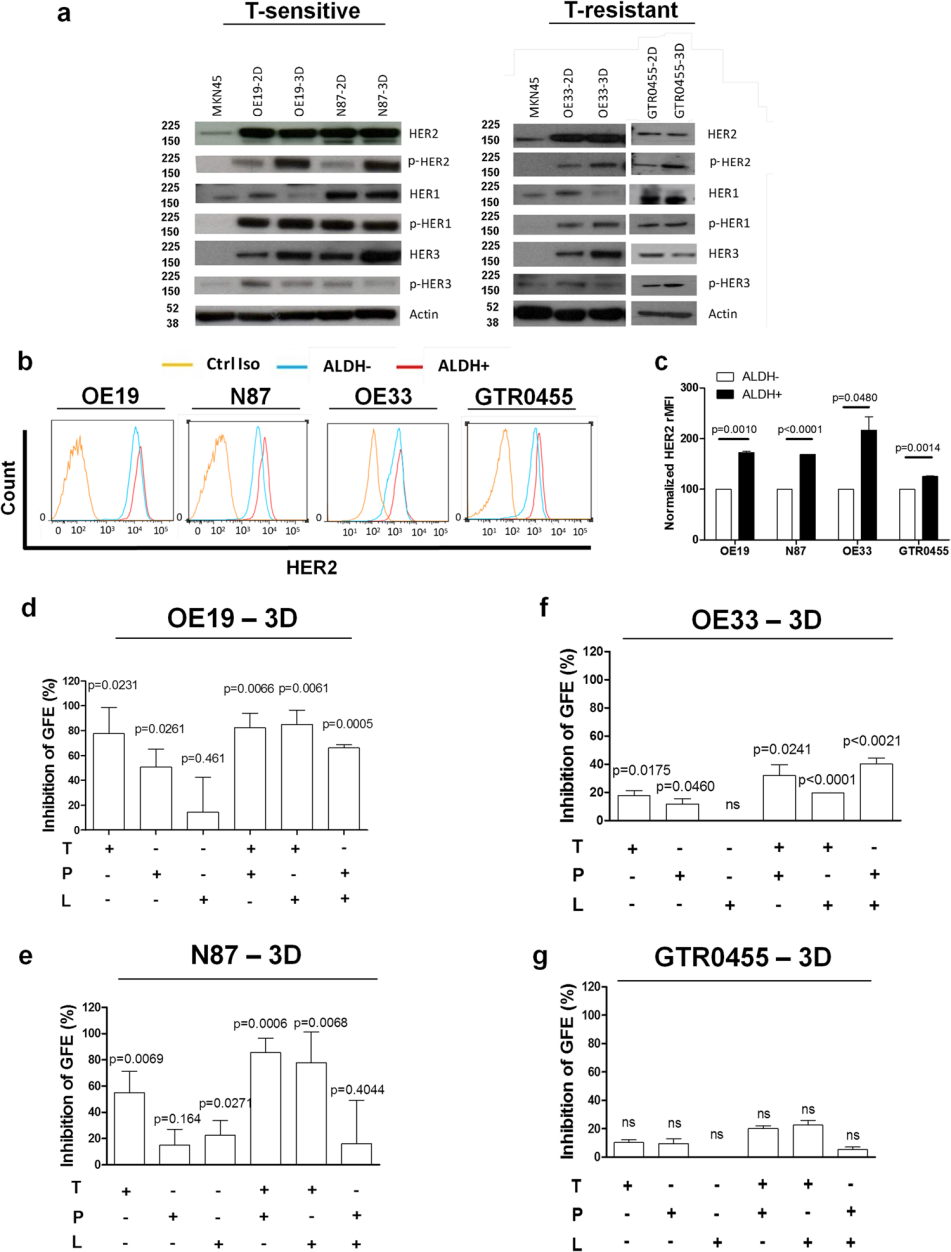
In vitro modulation of HER receptor expression/activation in HER2+ GC cell lines and inhibition of GFE (%) in matched HER2 + GCSC subsets according to treatment with anti-HER2 agents. **a**, Western blot analyses of HER1, HER2, and HER3 and their activated forms (pHER) in HER2+ GC cell lines cultured in adherent (2D) or gastrosphere-promoting (3D) conditions. Western blot analysis of MKN45 GC protein extract was used as an internal negative control, and only very low levels of basal HER2 and HER1 were detected. Actin was used to normalize protein loading. Autoradiographs of the left and right panels were acquired at different exposure times to obtain optimal image resolution. **b** and **c**, Evaluation of HER2 protein levels by FACS analyses (relative median fluorescence intensity [rMFI]) in the indicated cell lines gated for ALDH + cells versus the matched ALDH- counterpart. The values were normalized to HER2 expression in ALDH- cells. Column bars, mean ± SEM (n = 3). Significance was calculated by a two-tailed paired t test. **d-g**, Inhibition of GFE (%), normalized to internal controls (dilution vehicle of each drug: 0,9% NaCl for T and P; DMSO for L; T + L and P + L), in OE19 (**d**), N87 (**e**), OE33 (**f**), and GTR0455 (**g**) cells treated in 3D conditions for 7 days with T (20 μg/ml), P (30 μg/ml) and L (10 nM) and their combinations (T + P; T + L; T + P). Column bars, mean ± SEM (n = 3). Significance was calculated by a two-tailed paired t test

The basal and activation status of HER2 together with HER1 and HER3, two other members of the epidermal growth factor receptor family, was analyzed by Western blotting under reducing conditions in protein extracts from human T-sensitive (OE19 and N87) [36] and T-resistant (OE33 and GTR0455) [37, 38] HER2+ GC cells cultured either in adherent (2D) or gastrosphere-promoting (3D) conditions (Figs. 1a and S1). Specifically, we found that while basal HER2 expression levels were not altered by culture conditions, the amount of activated HER2 (p-HER2) was significantly increased in all the cell models cultured in 3D *versus* 2D conditions (Fig. 1a) as evaluated by densitometric analyses (Fig. S2). Conversely, the activation levels of EGFR (p-HER1) were not modified by culture conditions (Figs. 1a and S2). HER3 expression increased in cells grown in 3D *versus* their matched 2D counterparts, but no marked difference in its activation status was observed between 2 and 3D culture conditions (Figs. 1a and S2).

To verify whether HER2 was enriched in GCSCs *versus* the matched bulk cell counterparts, GC cells were gated for their reactivity to ALDH [12], a biomarker shared by CSCs of different epithelial oncotypes (Fig. 1b). Both ALDH + and ALDH- cell subsets were analyzed for HER2 expression by multiparametric FACS analysis (Fig. 1b and c). HER2 was expressed at significantly higher levels in the ALDH + cell compartment of all the tested cells compared with their matched ALDH- cell counterparts (Fig. 1b and c), thus revealing that increased HER2 levels preferentially occur in HER2+ GC cells with a stem phenotype. To examine whether the specific pharmacological inhibition of HER2 also targeted the GCSC compartment, HER2+ GC cells were cultured under 3D promoting conditions in the presence of T (20 µg/ml), lapatinib (L, 10 nM), pertuzumab (P, 10 µ g/ml) and their dual combinations (T + P; T + L; P + L) to evaluate gastrosphere forming efficiency (GFE %) (Fig. 1d-g). Specifically, T demonstrated a significant capability to inhibit the GFE % in T-sensitive cells, while P significantly inhibited GFE (%) only in OE19 cells (Fig. 1d and e). Conversely, L was significantly less active. Dual anti-HER2 combinations resulted in significantly impaired GFE (%) in OE19 cells (Fig. 1d), whereas only the two T-based blockades were able to significantly inhibit N87 GFE, and P + L was not effective (Fig. 1e). OE33 gastrospheres were refractory to the therapeutic effects exerted by L tested alone compared to T and P tested in monotherapy (P < 0.05) (Fig. 1f). The different dual anti-HER2 combinations were significantly more effective than each agent tested alone (from P < 0.05 to P < 0.0001) (Fig. 1f), although the biological/therapeutic effect remained limited due to intrinsic resistance. In keeping with reported data [38], GTR0455 gastrospheres were refractory to each monotherapy and each dual anti-HER2 drug combination tested (Fig. 1g).

Overall, these findings reveal the involvement of HER2 in the HER2+ GCSCs of all the tested GC cell lines regardless of their susceptibility to anti-HER2 agents. Additionally, since neither anti-HER2 monotherapy regimens nor the different dual HER2 blockade combinations completely inhibit the GFE (%), our findings imply that HER2+ GCSCs may express altered levels of other central molecules whose activity is responsible for HER2+ GCSC survival and proliferation.

### HER2+ GCSC compartment expresses high levels of FASN

**Fig. 2:**
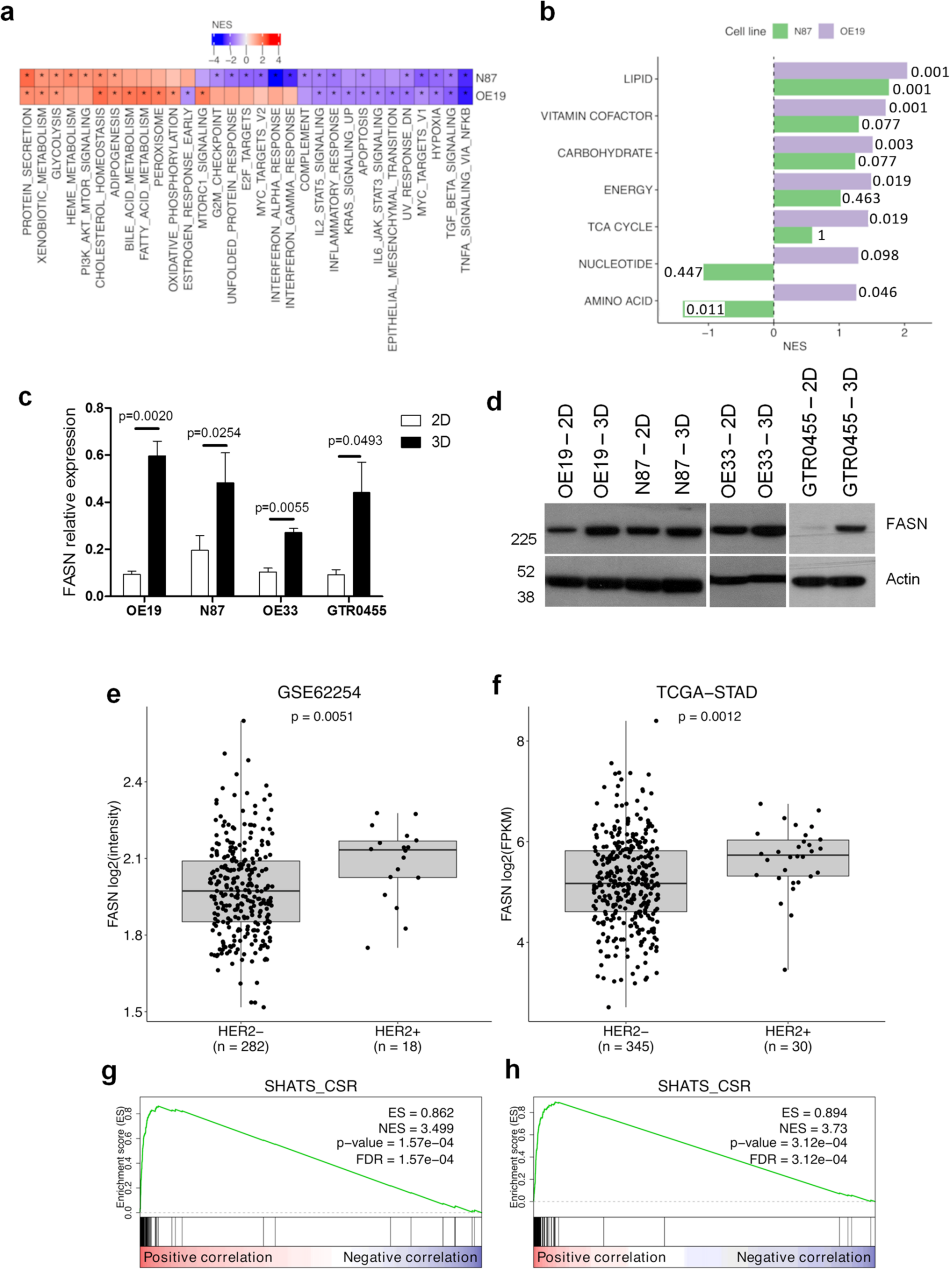
FASN expression levels in preclinical and clinical HER2+ GC models and enrichment of stemness-related genes in FASN-overexpressing HER2+ GC cases. **a**, Heatmap showing the normalized enrichment scores (NES) from GSEA comparison between N87 and OE19 cell lines cultured in 3D versus 2D conditions. Hallmark gene sets with FDR < 0.05 in at least one cell line are represented. Positive and negative values of NES indicate enrichment in HER2+ GC cell lines cultured in 3D and 2D conditions, respectively. *: FDR < 0.05. **b**, Bar plot of GSEA NES for metabolic subtype signatures from Peng X et al. (31) in the two cell lines. Positive and negative values of NES indicate enrichment in 3D and 2D cell lines, respectively. FDR values are also reported. **c**, FASN relative expression measured by qRT‒PCR in the indicated cell lines cultured in 2D and 3D conditions. Column bars, mean ± SEM (n = 4). Significance was calculated by a two-tailed paired t test. **d**, Western blotting analyses of OE19, N87, OE33 and GTR0455 protein extracts probed with anti-FASN antibody. Actin was used to normalize protein loading. E and F, Box plot of FASN expression according to ERBB2 (HER2) status in GSE62254 (**e**) and TCGA (**f**) GC gene expression datasets. Significance was calculated by the Wilcoxon test. **g** and **h**, GSEA results for the stemness SHATS-CSR signature in GSE62254 (**g**) and TCGA (**h**) gene expression data ranked according to correlation with FASN. The green line shows the distribution of the running enrichment score (ES) along the ranked gene list. Vertical black lines show the position of the genes of the SHATS-CSR signature in the ranked gene list

To identify new potential druggable markers whose expression is enriched in HER2+ GCSCs, we compared the gene expression profiles of OE19 and N87 cell lines cultured in 3D *versus* 2D conditions. We detected a positive enrichment of several metabolic gene sets in HER2+ 3D cells, particularly those related to FA metabolism and cholesterol homeostasis (Fig. 2a), by gene set enrichment analysis (GSEA). To investigate the modulation of metabolism-related processes, we performed GSEA using gene signatures specific for metabolic cancer subtypes [32]. We found that lipid metabolism was the only metabolic signature significantly enriched in both cell lines cultured in 3D conditions (Fig. 2b). By leading-edge analysis (Supplementary Table 1), we selected the genes that contributed the most to the enrichment of these lipid metabolism-related gene sets. Intersection of the leading-edge lists for the two cell lines led to the identification of five key genes (ACAT2, FASN, HSD17B7, IDI1, and NSDHL). We focused our attention on the FASN gene, the central metabolic multifunctional enzyme involved in “de novo” biosynthesis of FAs, because of its overexpression and hyperactivity in most human malignancies and the extensive literature already reported in HER2+ BC models [20–29]. To sustain GEP data, we performed qRT‒PCR analysis and found that the FASN gene was significantly upregulated in N87 and OE19 3D cell cultures *versus* their matched 2D counterparts (from P < 0.05 to P < 0.005) (Fig. 2c).

The same pattern was also observed in OE33 (P < 0.01) and GTR0455 (P < 0.05) cell models (Fig. 2c), suggesting that the FASN gene is a good therapeutic cotarget in HER2 + GCSCs. Similar results were also obtained by Western blot analysis in all HER2+ GC protein extracts (Fig. 2d).

To corroborate these results, we interrogated two public GC gene expression datasets, GSE62254 and TGCA-STAD (Fig. 2e and f). HER2+ and HER2- cases were sorted according to Erbb2 (HER2) expression (Fig. S3). In silico analyses of these two genome datasets revealed that FASN expression was significantly upregulated (P < 0.005) in HER2+ *versus* HER2- GC cases (Fig. 2e and f). To evaluate the association between an enhanced stem-like phenotype and FASN gene expression levels, we performed GSEA in the GSE62254 and TGCA-STAD GC datasets using an established stemness gene signature (consensus stemness ranking [CSR] signature- [33]). We observed that in both datasets, this signature was strongly enriched for genes positively correlated with FASN (Fig. 2g and h). Overall, these data provide clear evidence of significant FASN upregulation in human HER2+ GC cases and correlation with stem-like features.

### Anti-HER2 treatments induce FASN upregulation in preclinical and clinical HER2+ GC models

**Fig. 3:**
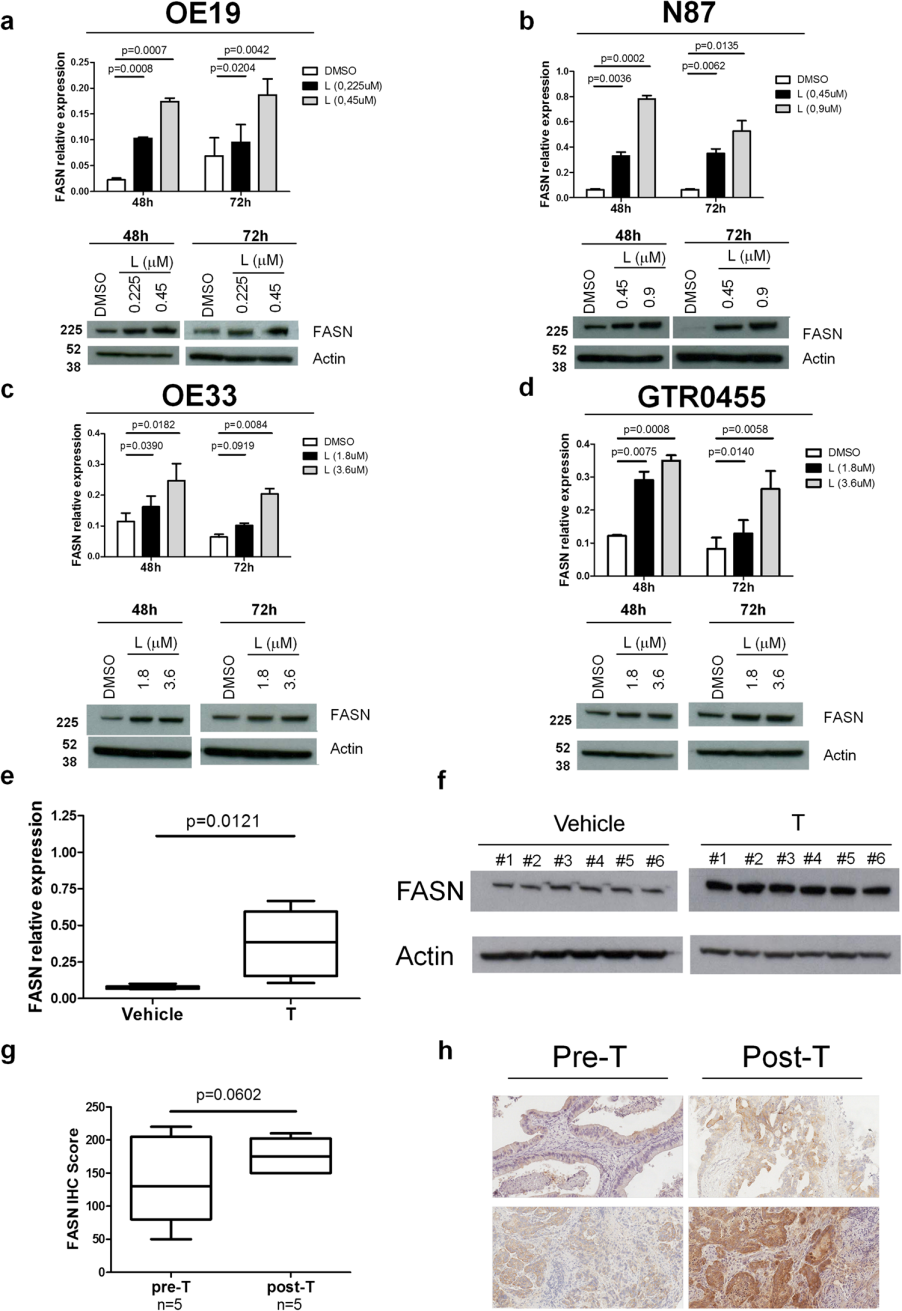
FASN expression levels in HER2+ GC cell lines and patients and GTR0455-implanted animals according to anti-HER2 agent treatment. **a-d**, FASN expression evaluated by qRT‒PCR and Western blotting in OE19 (**a**), N87 (**b**), OE33 (**c**) and GTR0455 (**d**) cells treated with L or DMSO for 48 h or 72 h. Blots were probed with anti-FASN or actin (loading control) antibodies. Actin was used to normalize protein loading. Autoradiographs were acquired at different exposure times to obtain optimal image resolution. **e** and **f**, FASN expression evaluated by qRT‒PCR (**e**) and Western blotting (**f**) in GTR0455 xenografts. NOD-SCID mice (n = 6/group) implanted with T-resistant GTR0455 cells were treated with T (30 mg/kg) or vehicle. Box plots, means ± SEMs. Significance was calculated by a two-tailed unpaired t test. Blots were probed with anti-FASN or actin (loading control) antibodies. Autoradiographs were acquired at the same exposure times. **g**, Box plots of H-score values of FASN IHC pre- and post-T therapy (n = 5); significance was calculated by a two-tailed paired t test. **h**, Images of FASN IHC in two pairs of pre- (on the left) and posttherapy (on the right) FFPE tumor samples, showing a diffuse increase in cytoplasmic immunoreactivity (20 × magnification)

To evaluate the effects of anti-HER2 treatments on FASN expression in HER2+ GC models, we tested its expression in HER2+ GC cell lines and in GTR0455 xenotransplants at baseline and upon incubation with L to block HER2 activity and in human specimens treated or not with T (Fig. 3). Specifically, OE19, N87, OE33 and GTR0455 cells were incubated with previously titrated proper concentrations of L (Fig. S4) for 48 h and 72 h. In vitro treatment with L proportionally induced enhanced expression of FASN in all cell models, both at the transcriptional and protein levels (Fig. 3a-d), as also validated by the densitometric analysis (Fig. S5), supporting the hypothesis that the inhibition of HER2 signaling could positively affect FASN expression.

To examine in vivo whether the treatment of T-resistant HER2+ GC cells with T can affect the expression of FASN, GTR0455 PDXs were administered with T (30 mg/kg) or, as an internal control, the diluent (vehicle). Analyses performed on GTR0455 tumors, collected when the volume reached approximately 1500 mm^3^, showed that FASN was significantly increased in T-treated (n = 6) *versus* vehicle-treated counterparts (n = 6) both at the transcriptional (P < 0.05, Fig. 3e) and protein levels (Fig. 3f). The expression of FASN was also analyzed by IHC in matched pre- and post-T treated biopsies from human HER2+ mGC (Fig. 3g and h). Consistent with all preclinical findings, we observed a coherent increase in FASN protein at the clinical level in post-T cases *versus* their pre-T samples (Fig. 3g). Indeed, IHC staining of matched cases showed an overall increase in cytoplasmatic tumor cell immunoreactivity, with median H-scores of 110 (IQR: 82.5–182.5) and 165 (IQR: 150–195) in pre- and post-treatment samples, respectively (p = 0.0602) (Fig. 3h). Altogether, our findings show a consistent enhancement of FASN expression upon T treatment.

### Inhibition of FASN expression/activity targets GCSCs in HER2+ GC cell lines

**Fig. 4:**
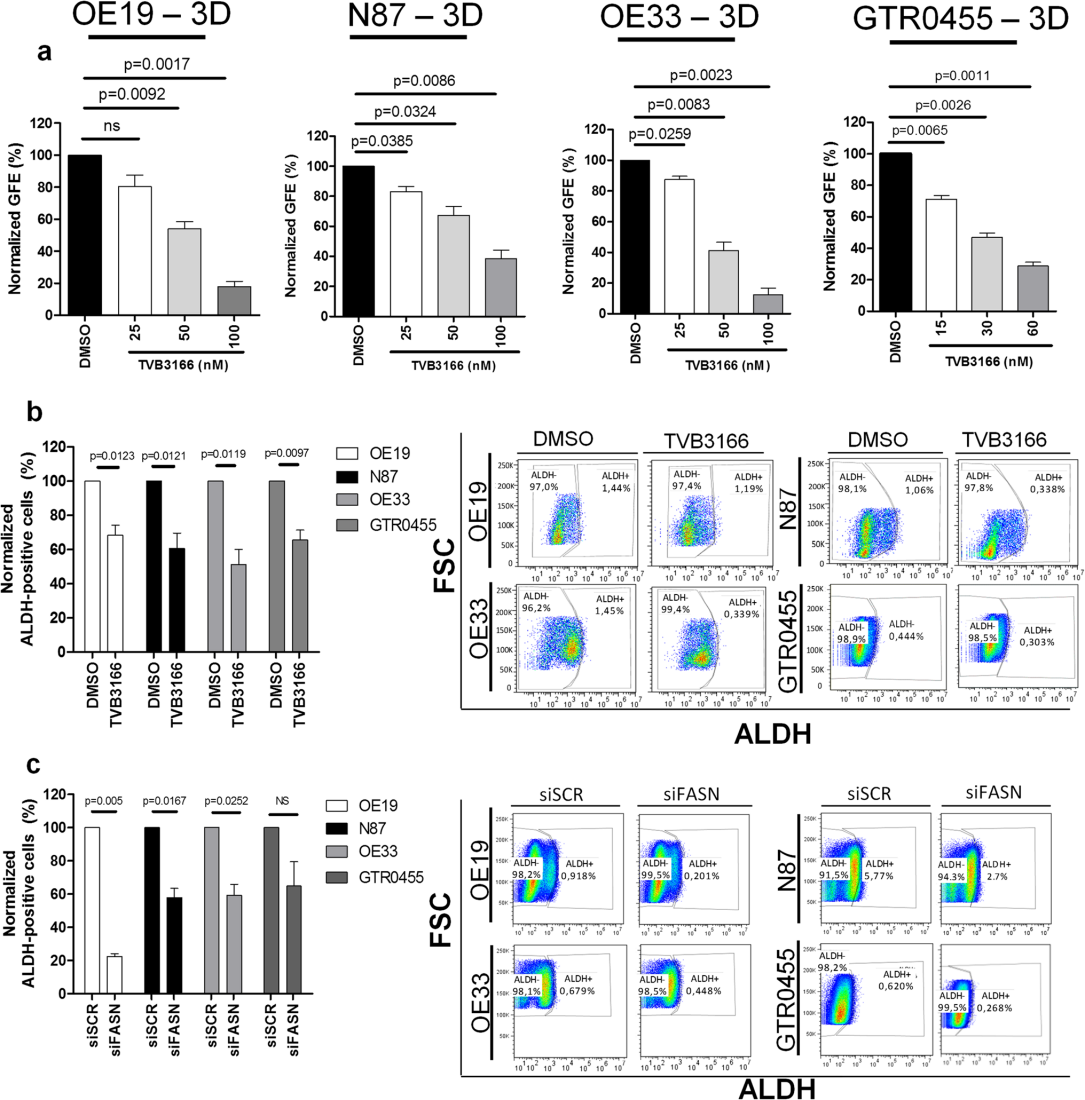
Treatment of HER2+ gastrospheres with the specific anti-FASN agent TVB3166 and targeting of ALDH + cells upon pharmacological and genetic inhibition of FASN activity. **a**, Inhibition of GFE % in OE19, N87, OE33 and GTR0455 cells treated with different concentrations of TVB3166. The number of formed gastrospheres was evaluated after 7 days of treatment. The values were normalized to the GFE (%) of cells cultured with the dilution vehicle of TVB3166 (DMSO). Column bars, means ± SEMs (n = 3). Significance was calculated by a two-tailed paired t test. **b** (left panel), % of ALDH + OE19, N87, OE33 and GTR0455 cells treated with TVB3166 (20 mM) for 48 h. The values were normalized to the percentage of ALDH + cells in DMSO-treated cells. The results are presented as the mean ± SEM (n = 3). Significance was calculated using a two-tailed paired t test; (right panel). Representative dot plots showing the % of ALDH + and ALDH- cells in OE19, N87, OE33 and GTR0455 cells treated with DMSO or TVB3166. **c** (left panel), % of ALDH + OE19, N87, OE33 and GTR0455 cells transfected with scramble control siRNA (siSCR) or specific FASN siRNA (siFASN) for 48 h. The values were normalized to the percentage of ALDH + cells in engineered siSCR cells. The results are presented as the mean ± SEM (n = 3). Significance was calculated using a two-tailed paired t test. (right panel), Representative dot plots showing the % of ALDH + and ALDH- cells in OE19, N87, OE33 and GTR0455 cells engineered with siSCR or siFASN

We tested the antitumor effects of TVB3166, a selective commercial FASN inhibitor [40], in OE19, N87, OE33 and GTR0455 gastrospheres.. TVB3166, tested at different concentrations, impaired in a dose-dependent manner the GFE (%) of GCSCs derived from the T-sensitive and T-resistant cell lines compared with vehicle (DMSO)-treated gastrospheres, with a statistical significance ranging from P < 0.05 to P < 0.005 (Fig. 4a). To further sustain the capability of TVB3166 to target HER2+ GCSCs, we examined its therapeutic effects on the ALDH + cell compartments derived from all the cell models. We observed that 48 h of treatment with a nontoxic concentration of TVB3166 (Fig. S6) led to a significant decrease in ALDH + cells in all the cell lines (P < 0.05) (Figs. 4b and S7). Notably, a significantly relevant reduction in ALDH + cells was also detected upon transient FASN silencing (siFASN) *versus* each control siRNA (siSCR) (P < 0.05) (Fig. S8a and b), even for the highly resistant GTR0455 cells that showed a decreasing trend (P = 0.138) (Figs. 4c and S9). Altogether, our results support the potential candidacy of FASN as a novel metabolic druggable target that is upregulated in the HER2+ GCSC compartment.

**Fig. 5:**
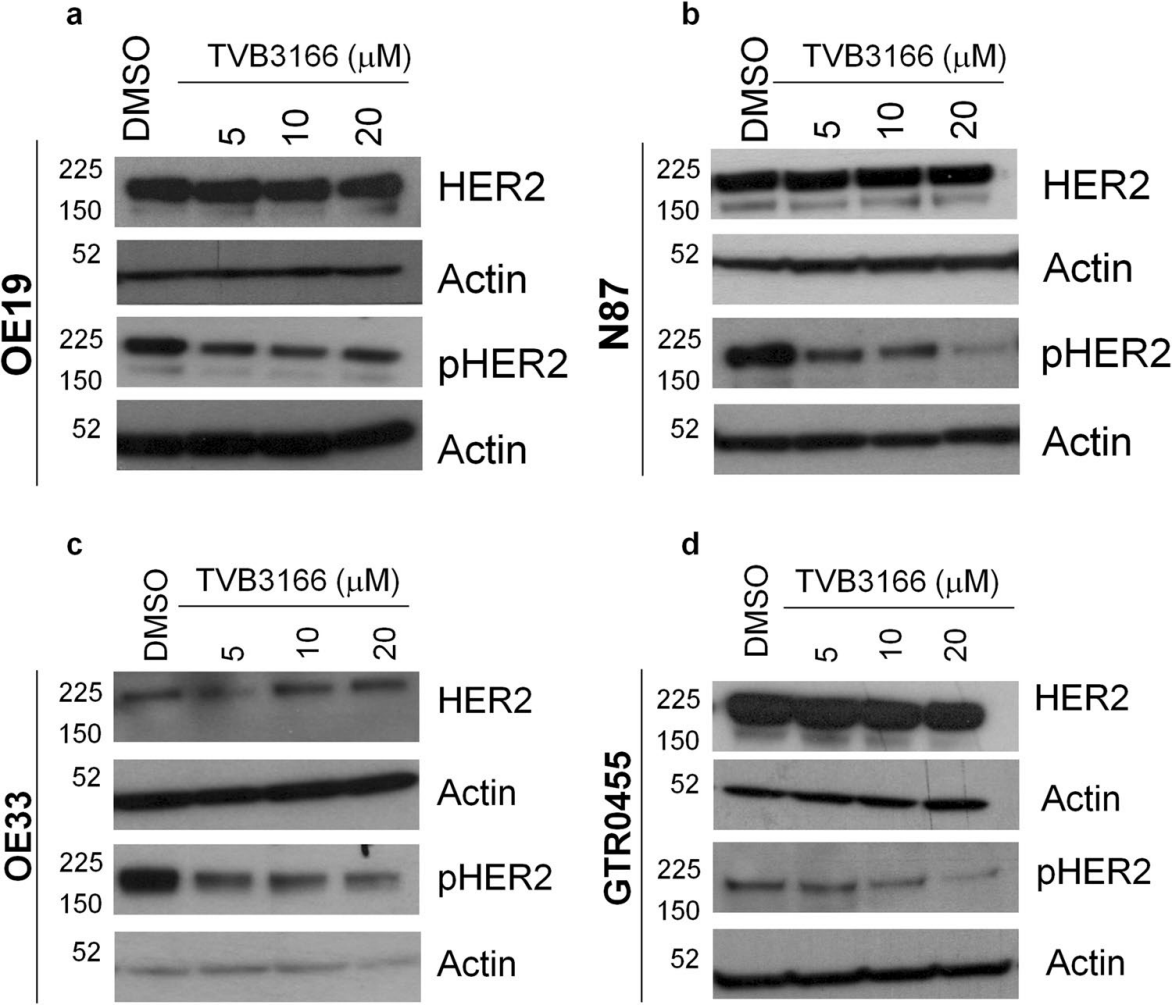
Anti-FASN treatment decreases HER2 activation in HER2+ GC cell lines. **a-d.** Western blotting analyses of OE19 (**a**), N87 (**b**), OE33 (**c**) and GTR0455 (**d**) protein extracts from cells treated with DMSO or TVB3166 at different concentrations (5 mM, 10 mM and 20 mM) for 72 h. Blots were probed with anti-HER2 and anti-pHER2 antibodies. Actin was used to normalize protein loading. Autoradiographs were acquired at different exposure times to obtain optimal image resolution

To investigate the link between FASN activity and HER2-driven GCSC maintenance in the HER2 + GC, we analyzed the effects of pharmacological FASN inhibition on the expression and activation status of HER2. OE19, N87, OE33, and GTR0455 cells were treated with different nontoxic concentrations of TVB3166. Western blotting analysis (Fig. 5a-d) showed that TVB3166 did not exert any evident effect on basal HER2 expression, whereas, a more or less marked reduction in pHER2 was observed in all the cell lines at every drug concentration tested. These results strongly sustain that FASN inhibition impairs, to some extent, HER2 activation and, in turn, negatively regulate HER2+ GC and GCSCs maintenance.

### Dual blockade of HER2 and FASN overcomes resistance to anti-HER2 agents in HER2+ GCSCs

**Fig. 6:**
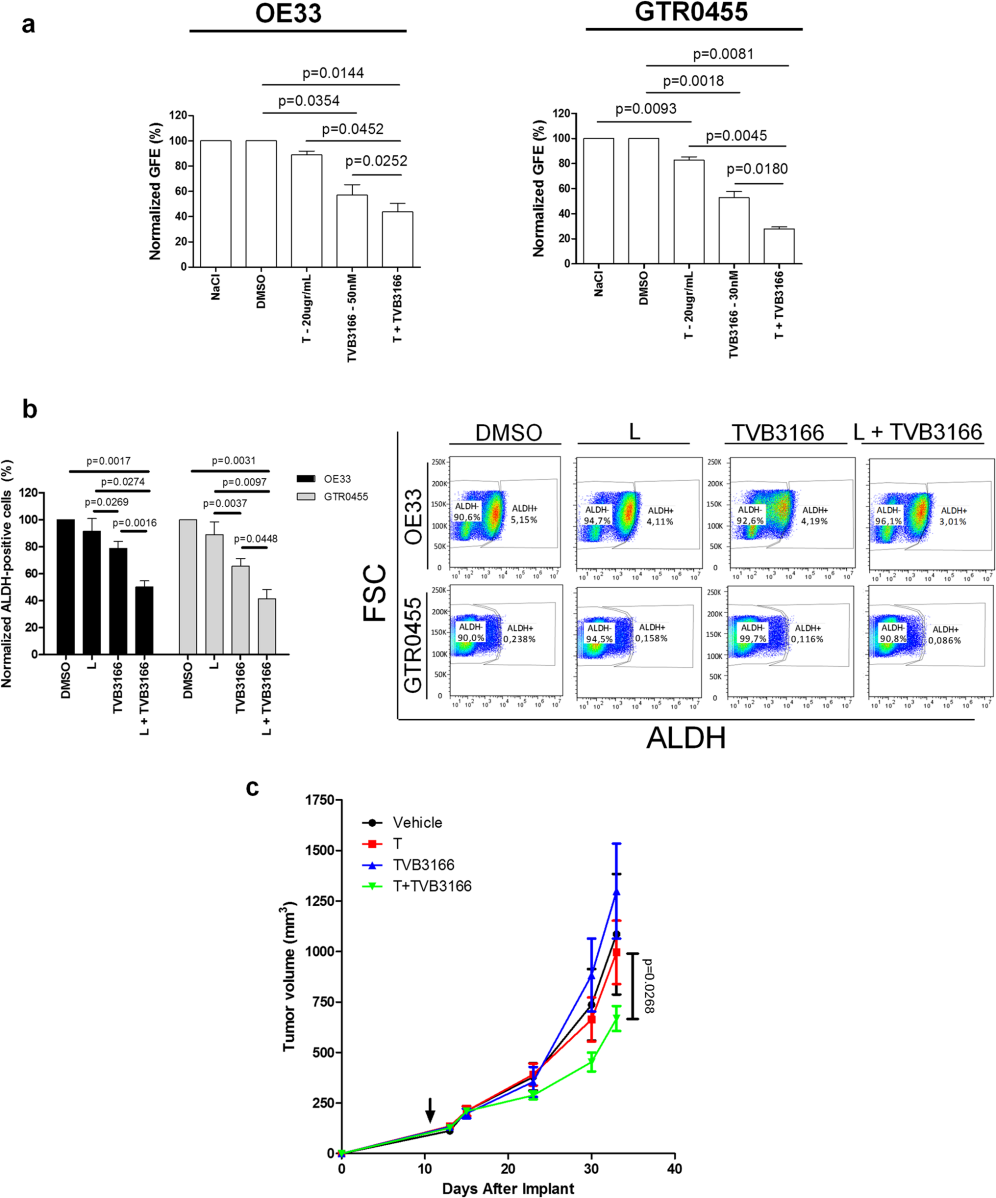
Therapeutic activity of dual HER2 and FASN blockade evaluated in T-resistant HER2+ 3D cell models and animals injected with GTR0455 cells. **a**, GFE % of T-resistant OE33 and GTR0455 cells lines treated with NaCl 0.9%, DMSO, T, TVB3166 and T + TVB3166. The number of formed gastrospheres was evaluated after 7 days of treatment. The values were normalized to the GFE (%) of cells cultured with vehicles. Column bars, means ± SEMs (n = 4). Significance was calculated by a two-tailed paired t test. **b** (left panel), % of ALDH + OE33 and GTR0455 cells treated with L (0.9 mM), TVB3166 (10 mM) and their combination (L + TVB3166) for 48 h. Values were normalized to the % of ALDH + cells in DMSO-treated samples. The results are presented as the mean ± SEM (n = 3). Column bars, mean ± SEM (n = 4). Significance was calculated by a two-tailed paired t test. **b** (right panel), representative dot plots showing the % of ALDH + and ALDH- cells in OE33 and GTR0455 cells treated with DMSO, L, TVB3166 or L + TVB3166. **c**, Tumor growth curve of GTR0455 cells subcutaneously implanted in NOD SCID mice followed by treatment with TVB3166, T, or their combination (T + TVB3166). The arrow indicates the start of treatment. Data are presented as the mean ± SEM. Significance was calculated using two-way ANOVA followed by Bonferroni multiple comparisons correction

To elucidate whether FASN inhibition is a novel anticancer strategy to overcome CSC-mediated therapeutic resistance to anti-HER2 therapies, gastrospheres derived from OE33 and GTR0455 cell lines resistant to anti-HER2 agents [35, 38] were treated with TVB3166 (OE33: 50 nM; GTR0455: 30 nM) and T (20 µg/ml) in monotherapy and in combination (Fig. 6a). We found that TVB3166 plus T mediated a significant GFE (%) impairment versus each monotherapy regimen (from P < 0.05 to P < 0.005). These data support that the simultaneous metabolic blockade of FASN and HER2 can achieve more potent anti-GCSC effects than each monotherapy alone. Further, the same HER2+ GC cell lines were treated in 2D conditions with TVB3166 and L (instead of T known to have a reduced antitumor capacity in HER2+ cells cultured in 2D conditions), both in monotherapy and in combination, to evaluate the impact of these treatments on the % of gated ALDH + cells. The combination of TVB3166 plus L exhibited a significantly higher capability to decrease the percentage of ALDH + cells than any of the monotherapy treatments (Figs. 6b and S10) (from P < 0.05 to P < 0.005), supporting the hypothesis that the inhibition of FASN could ameliorate the benefits exerted by anti-HER2 biodrugs. To reinforce our speculation and corroborate the in vitro results, GTR0455 cells were injected subcutaneously into NOD-SCID mice, which were then randomized and treated with TVB3166, T or their combination. According to in vitro data, in vivo dual blockade of HER2 and FASN also slowed tumor growth compared to the administration of T or TVB3166 as a monotherapy (Fig. 6c).

## Discussion

Several successes achieved in patients with HER2+ BC have not been reproduced in patients with HER2+ GC, mainly owing to several biological differences, including the higher degree of heterogeneity of HER2 expression in GC and the coexistence of other genomic drivers beyond HER2 itself [41].

Since altered HER2 signaling has a central role in the maintenance/survival of breast CSCs [12] and despite the availability of limited data on HER2+ GC stemness [15], the addition of GCSC-targeted therapies to current treatment options may be a key step for improving patient outcomes [6, 42]. In this context, we found that the intrinsic sensitivity of the tested HER2+ GC cells to HER2 targeting is positively associated with the per se capability of anti-HER2 agents to impair gastrosphere formation; indeed, only low or no levels of GFE % inhibition were observed in T-resistant cell targets, confirming the existence of additional drivers supporting cancer stemness features.

Compelling evidence suggests that stem-like features can be acquired as a result of metabolic shifts, a phenomenon termed “metabostemness”, where the altered expression/activity of some key metabolic enzymes involved in glycolysis and/or lipid metabolism can act as oncogenic drivers, and their targeting may represent a successful strategy [43]. In particular, lipids execute fundamental roles in almost all cell activities, including cell‒cell recognition, signal transduction and energy supply. Accumulating data in the field of CSC metabolism provides evidence that these malignant cells rely heavily on the activity of enzymes involved in lipid metabolism to exert their peculiar tumorigenic properties [18, 44]. Accordingly, our present transcriptomic findings revealed that HER2+ GCSC cultures, compared with their matched 2D cell counterparts, exhibited a significant enrichment of genes included in lipid FA metabolism and cholesterol homeostasis pathways. Among the key five genes that mostly contributed to the enrichment of lipid metabolism gene sets in GCSCs, we focused our attention on the FASN gene. This was mainly due to the well-established bidirectional linkage occurring between FASN and HER2 in the context of BC [21] and the availability of selective inhibitors under clinical development in HER2+ BC. In addition, we considered the close involvement of FASN in antitumor therapeutic resistance, CSC maintenance/survival and cancer cell plasticity [19]. Globally, all these cell programs regulated by altered FASN expression heavily contributed to the assumption that FASN might function as a metabolic intermediate of oncogenesis linking energy, anabolism, carcinogenesis, and tumor invasiveness [20].

Notably, we found the existence of a significant positive correlation between an enrichment of GC stemness-related genes included in a stem cell-based signature [33] with FASN overexpression in HER2+ GC patients, thus suggesting that targeting FASN might be clinically relevant to arrest one key survival advantage imparted to CSCs by metabolic plasticity [18]. These findings fit well with a significant association reported between the overexpression of FASN and HER2 at the clinical level in a cohort of GC patients where those with concordant expression patterns of both proteins in GC tissues had a worse prognosis potentially due to their simultaneous and reciprocal activation [23]. Accordingly, the inhibition of FASN activity was also reported to affect CSC traits in glioma [45], pancreas [46] and breast [47] cancers. Different mechanisms have been proposed to explain the antitumor effects achieved by FASN blockade in the biological context of HER2+ BC [20, 48]. Similarly to already reported findings, we observed that the inhibition of FASN, whether via a specific agent or gene silencing/knockdown, significantly impaired GFE% of all the tested HER2+ GCSC populations, regardless of their susceptibility to anti-HER2 agents, probably via deactivation of HER2 signaling. Based on data already published in the HER2+ BC context [20], here we hypothesize that FASN inhibition in HER2+ GC cells impairs HER2 activation and, in turn, its downstream oncogenic signaling. This may happen through a potential alteration of phospholipids synthesis, strictly depending by FASN activity, with the consequent disruption of the lipid raft architecture on the plasma membrane, where the oncoprotein is mainy located, thus negatively impacting on HER2+ GCSC traits.

In addition, we observed that the pharmacological inhibition of HER2 signaling upregulated FASN expression in HER2+ GC models, leading us to speculate that the FA pathway is a key player in maintaining HER2+ GCSCs. Although these results appear counterintuitive in light of the reported existence of a positive feedback loop between HER2 and FASN activity (19), we hypothesize that HER2 inhibition could negatively impact glycolysis, a catabolic pathway directly controlled by HER2 signaling [49], thus leading to a metabolic shift to promote the use of endogenous FAs as energetic sources via FA β-oxidation (FAO). Accordingly, FASN activity has been reported to regulate FAO in colorectal cancer, promoting the oxidation of endogenous FAs to produce ATP [50].

Of note, we observed that dual FASN and HER2 blockade significantly increased in vitro and in vivo therapeutic benefits by decreasing the GFE% of T-refractory cell models and by reducing tumor growth in mice implanted with highly refractory GTR0455 GC cells *versus* T-based monotherapy. Although the specific functional mechanism(s) contributing to the increased therapeutic efficacy of dual FASN/HER2 blockade in HER2+ GC remain to be clearly elucidated, thus representing a limitation of this study, our present data demonstrated that targeting FASN in HER2+ GC may be a novel strategy to benefit patients with T resistance. The availability of a low-toxicity anti-FASN biodrug in a clinical setting makes FASN inhibition a promising therapeutic approach in combination with T to efficiently target HER2+ GCSCs and highly proliferating HER2+ GC cells. Moreover, it was previously shown that caveolin-1 depletion significantly increases the stability and availability of cell surface HER2, thus enhancing the binding of trastuzumab and improving its therapeutic efficacy [51]. We believe that a more complete understanding of the biochemical and molecular consequences of FASN and cholesterol inhibition might pave the way to future studies aimed to design a less cytotoxic dual lipid metabolic blockade via statins and TVB3166 in combination with anti-HER2 agents in HER2 + GC.

## Conclusions

Our data demonstrate that FASN upregulation represents a crucial hub in the maintenance of HER2+ GCSC population, and, accordingly, FASN inhibition determines a marked decrease of HER2 activation known to be an important regulator of HER2+ CSC maintenance. Consistently, we observed that the pharmacological dual blockade of HER2 and FASN targets CSCs more efficiently than monotherapies and reduced the in vivo growth of trastuzumab-resistant HER2+ GC cells. The overarching goal of our study is the design of a potential novel therapeutic strategy for advanced HER2+ GC, for instance, by cotargeting HER2 and FASN oncogenic/metabolic axis to eradicate both the root of cancer and the bulk of the tumor mass.

## Supplementary Information

Below is the link to the electronic supplementary material.

Fig. S1 Representative pictures of HER2+ GC cell lines OE19, N87, OE33 and GTR0455 cultured in adherent (2D – magnification 4X) or gastrosphere-promoting conditions (3D - magnification 10X). Spheres formed after 7 days of incubation. Crystal violet was used to stain cells attached to cell culture plates. (PNG 1400 kb)

Supplementary file1 (TIF 3415 KB)

Fig. S2 Densitometric analysis of basal HER1, HER2, and HER3 and their activated forms (p-HER) expression in OE19, N87, OE33 and GTR0455 cells cultured in 2D and 3D conditions evaluated by Western Blot shown in Fig. 1a. Protein extracts were separated by 4-12% gradient SDS-PAGE under reducing conditions. (PNG 160 000 kb)

Supplementary file2 (TIF 1415 KB)

Fig. S3 Distribution of ERBB2 (HER2) gene expression in GSE62254 and TCGA datasets. The dashed vertical line represents the cutoff used to stratify patients into HER2- and HER2+ groups. (PNG 231 000 kb)

Supplementary file3 (TIF 1451 KB)

Fig. S4 Antitumor growth activity of L in OE19, N87, OE33 and GTR0455 cells cultured in adherent 2D conditions and evaluated by a WST-1-cell cytotoxicity assay. Cells were grown for 24 h, 48 h and 72 h in the presence of increasing concentrations of L. The values were normalized to the growth of the cells incubated with DMSO. (PNG 108 600 kb)

Supplementary file4 (TIF 1053 KB)

Fig. S5 Densitometric analysis of FASN expression in OE19, N87, OE33 and GTR0455 cells after treatment with lapatinib (L) or DMSO evaluated by Western Blot shown in Fig. 3a-d. Protein extracts were separated by 4-12% gradient SDS-PAGE under reducing conditions. (PNG 122 000 kb)

Supplementary file5 (TIF 1225 KB)

Fig. S6 Antitumor growth activity of TVB3166 in OE19, N87, OE33 and GTR0455 cells cultured in adherent 2D conditions and evaluated by a WST-1 cytotoxicity assay. Cells were grown for 24 h, 48 h and 72 h in increasing concentrations of TVB3166. The values were normalized to the growth of the cells incubated with DMSO. (PNG 155 000 kb)

Supplementary file6 (TIF 1073 KB)

Fig. S7 Representative dot plots showing the DEAB control sample used as a background fluorescence signal for ALDEFLUOR™-stained OE19, N87, OE33 and GTR0455 cells treated with TVB3166 and DMSO. (PNG 569 000 kb)

Supplementary file7 (TIF 2392 KB)

Fig. S8 a, qRT‒PCR analyses of FASN expression evaluated in OE19, N87, OE33 and GTR0455 cell lines transiently transfected with siSCR or siFASN constructs. Columns bars, means ± SEMs (n=3). Significance was calculated by a two-tailed paired t test. b, Western blot analysis of FASN expression in OE19, N87, OE33 and GTR0455 cell lines transiently transfected with siSCR or siFASN constructs. Blots were probed with FASN antibody. Actin was used to normalize protein loading. (PNG 141 000 kb)

Supplementary file8 (TIF 973 KB)

Fig. S9 Representative dot plots showing the DEAB control sample used as the background fluorescence signal for ALDEFLUOR™-stained OE19, N87, OE33 and GTR0455 cells transfected with siSCR or siFASN primers. (PNG 585 000 kb)

Supplementary file9 (TIF 2203 KB)

Fig. S10 Representative dot plots showing the DEAB control sample used as a background fluorescence signal for ALDEFLUOR™-stained OE33 and GTR0455 cells treated with DMSO, L, TVB3166 and L+TVB3166. (PNG 341 000 kb)

Supplementary Table 1 Results of GSEA in the comparison between 3D versus 2D cell lines (TIF 1519 KB)

Supplementary file11 (XLSX 35 KB)

## Data Availability

All data related to this study are available within the article and its Supplementary information files. Gene expression data for this study are available from the Gene Expression Omnibus (GEO) with accession number GSE212442.
