# Supplementary figures and images for: Fatty acid synthase as a new therapeutic target for HER2-positive gastric cancer
Source: Cell Oncol (Dordr). 2023 Feb 8;46(3):661–76. doi: 10.1007/s13402-023-00769-x (PMC10205874; doi:10.1007/s13402-023-00769-x)

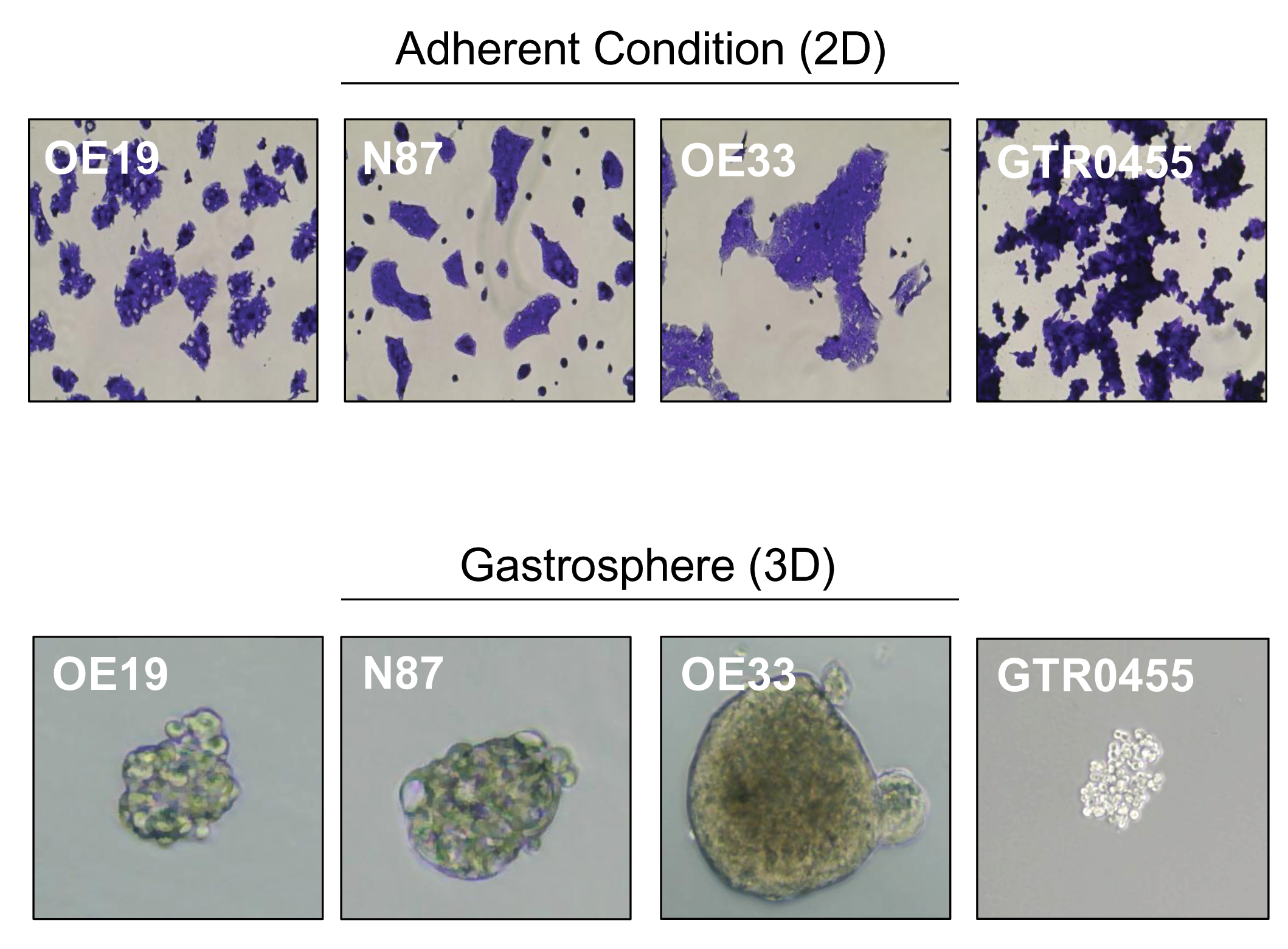

Supplement: Supplementary file 1 — Representative pictures of HER2+ GC cell lines OE19, N87, OE33 and GTR0455 cultured in adherent (2D – magnification 4X) or gastrosphere-promoting conditions (3D - magnification 10X). Spheres formed after 7 days of incubation. Crystal violet was used to stain cells attached to cell culture plates. (PNG 1400 kb) [file 13402_2023_769_Fig7_ESM.png]

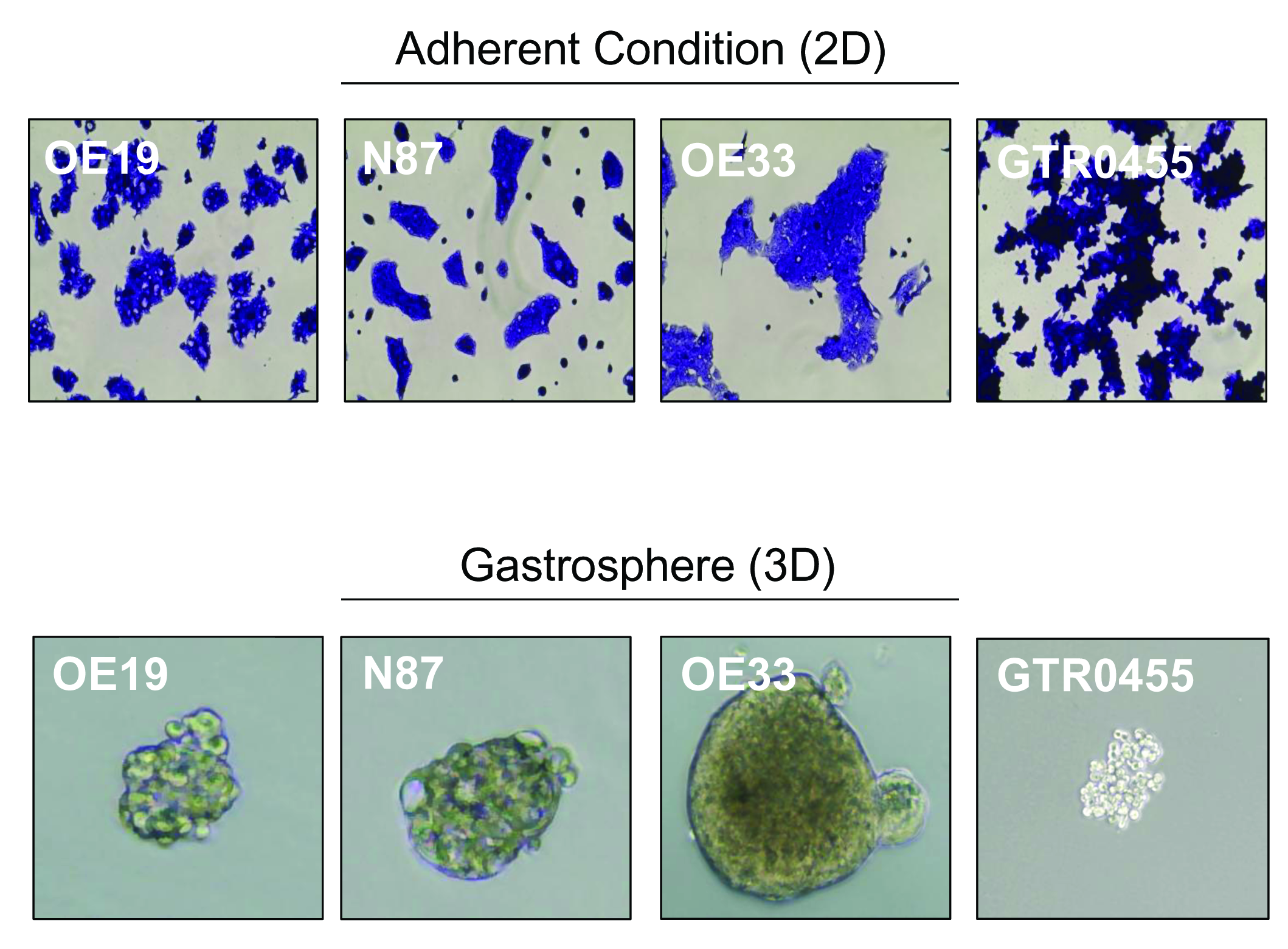

Supplement: Supplementary file 2 — Supplementary file1 (TIF 3415 KB) [file 13402_2023_769_MOESM1_ESM.tif]

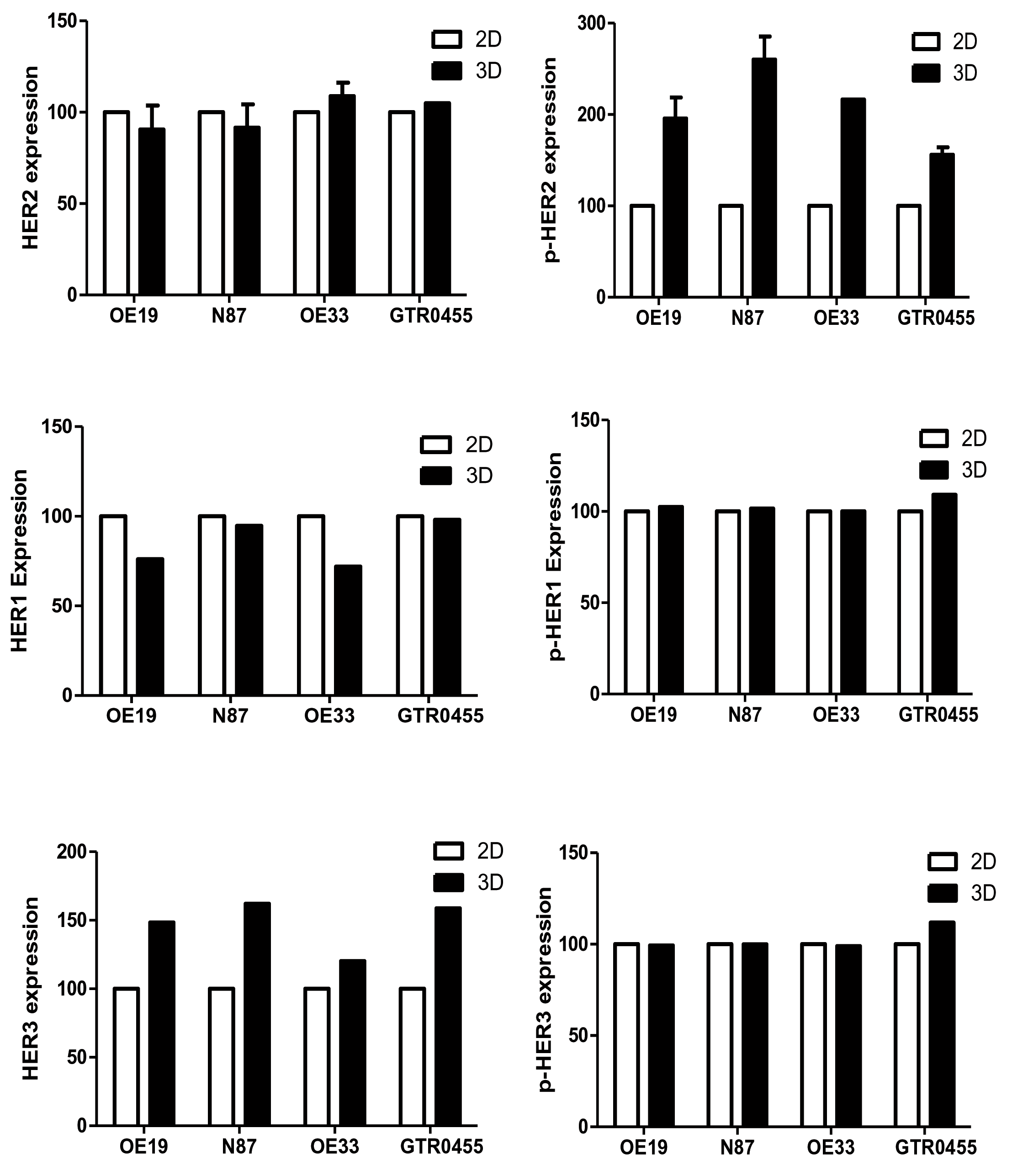

Supplement: Supplementary file 3 — Densitometric analysis of basal HER1, HER2, and HER3 and their activated forms (p-HER) expression in OE19, N87, OE33 and GTR0455 cells cultured in 2D and 3D conditions evaluated by Western Blot shown in Fig. 1a. Protein extracts were separated by 4-12% gradient SDS-PAGE under reducing conditions. (PNG 160 000 kb) [file 13402_2023_769_Fig8_ESM.png]

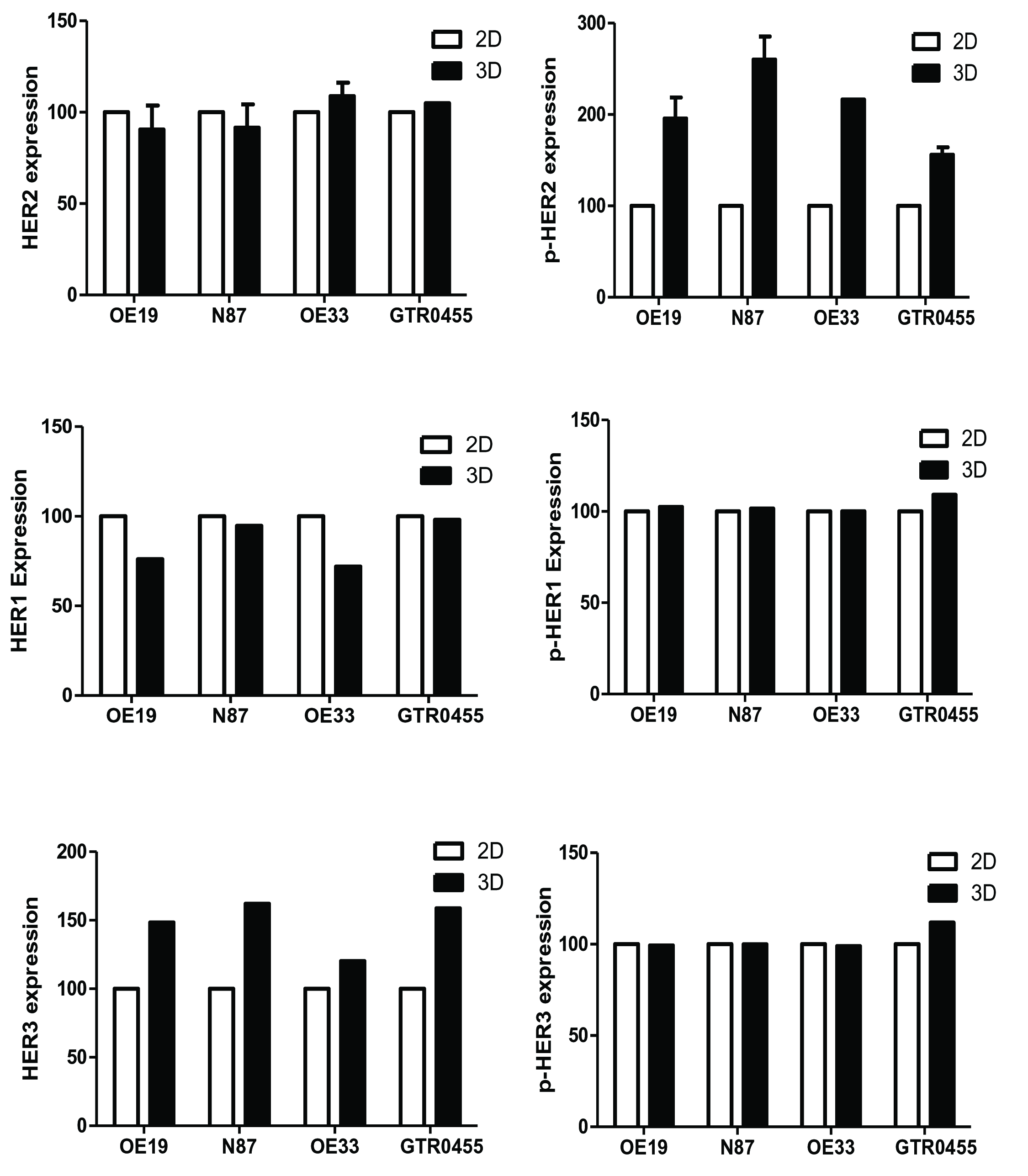

Supplement: Supplementary file 4 — Supplementary file2 (TIF 1415 KB) [file 13402_2023_769_MOESM2_ESM.tif]

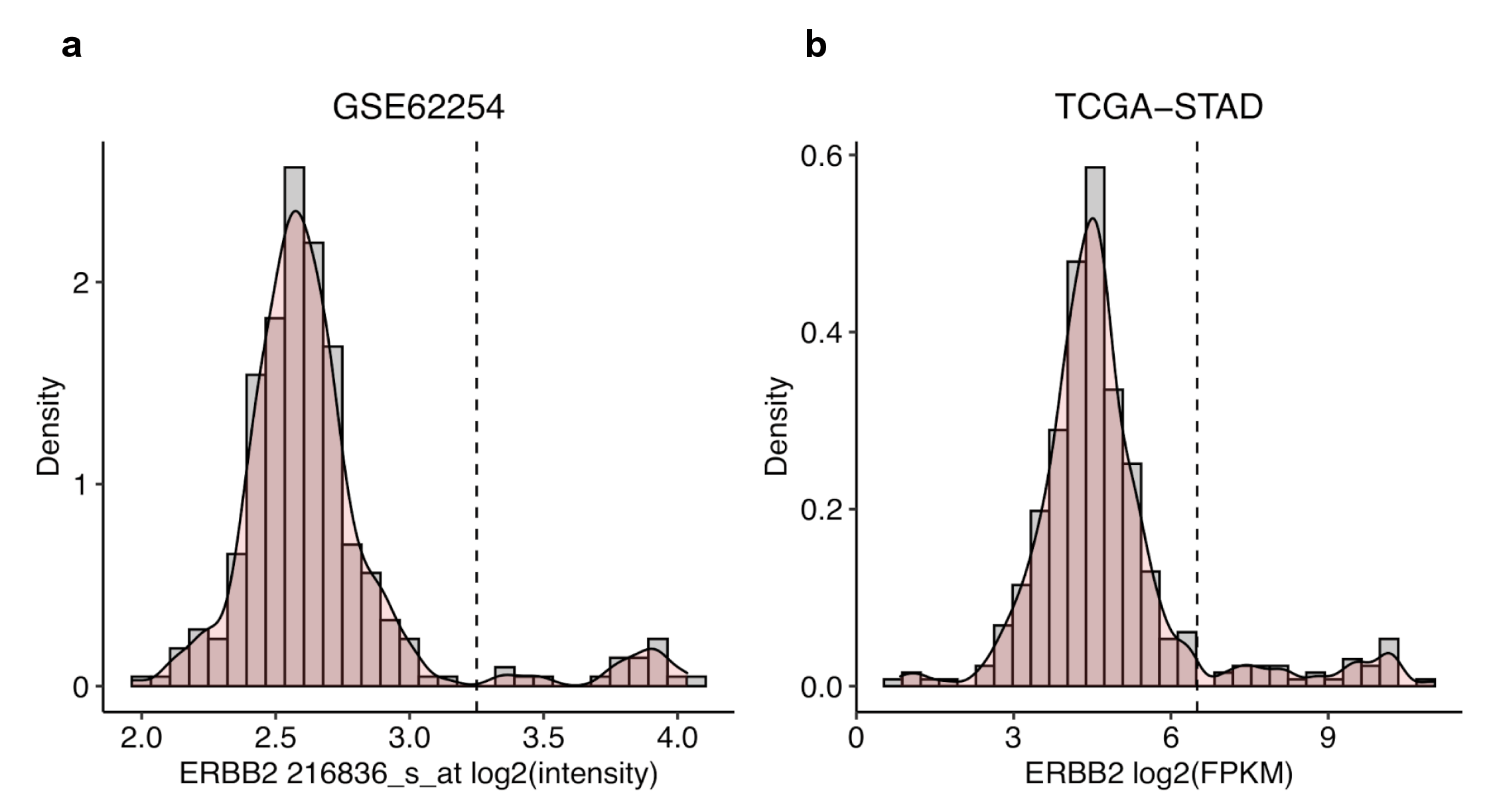

Supplement: Supplementary file 5 — Distribution of ERBB2 (HER2) gene expression in GSE62254 and TCGA datasets. The dashed vertical line represents the cutoff used to stratify patients into HER2- and HER2+ groups. (PNG 231 000 kb) [file 13402_2023_769_Fig9_ESM.png]

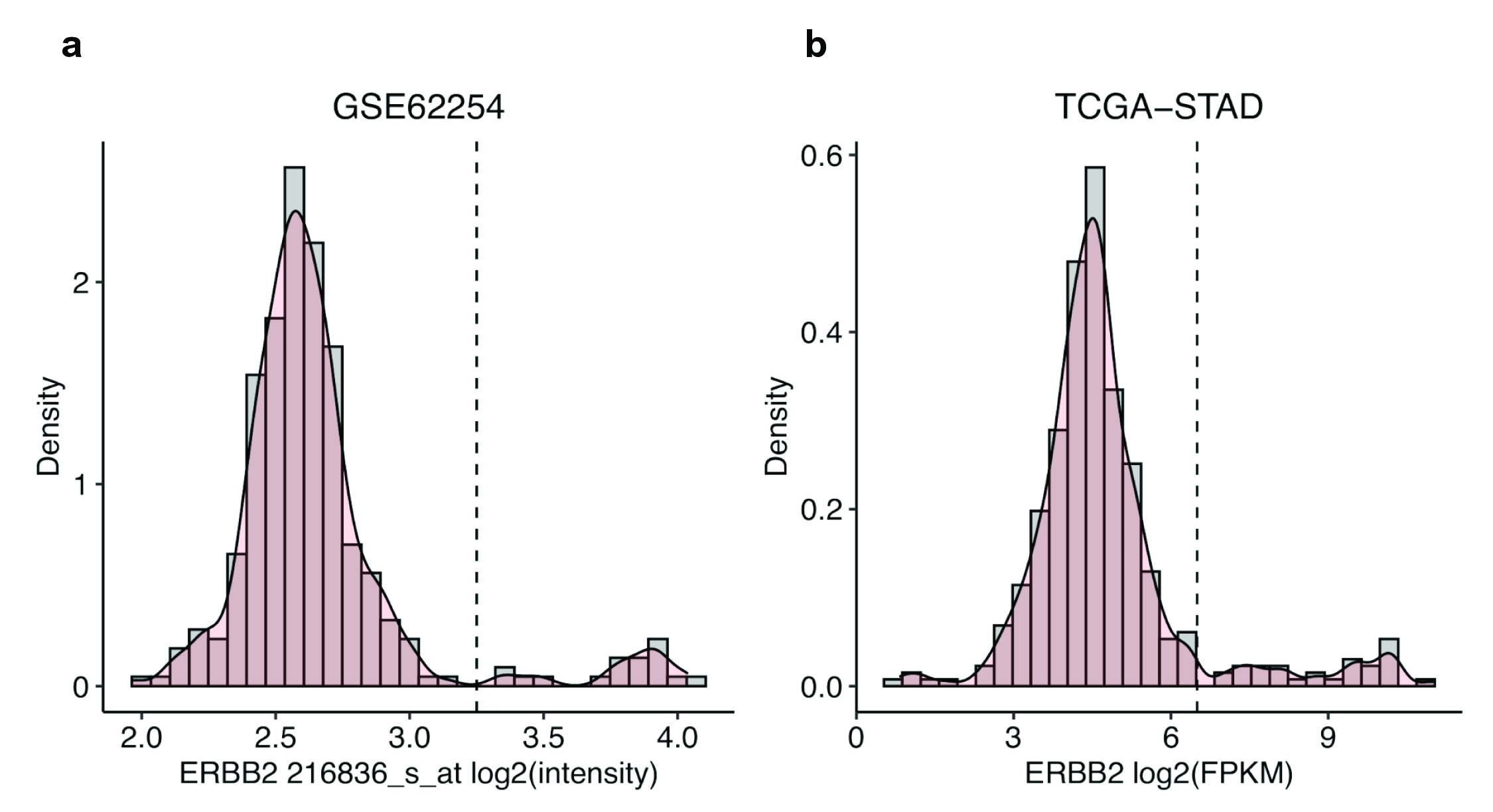

Supplement: Supplementary file 6 — Supplementary file3 (TIF 1451 KB) [file 13402_2023_769_MOESM3_ESM.tif]

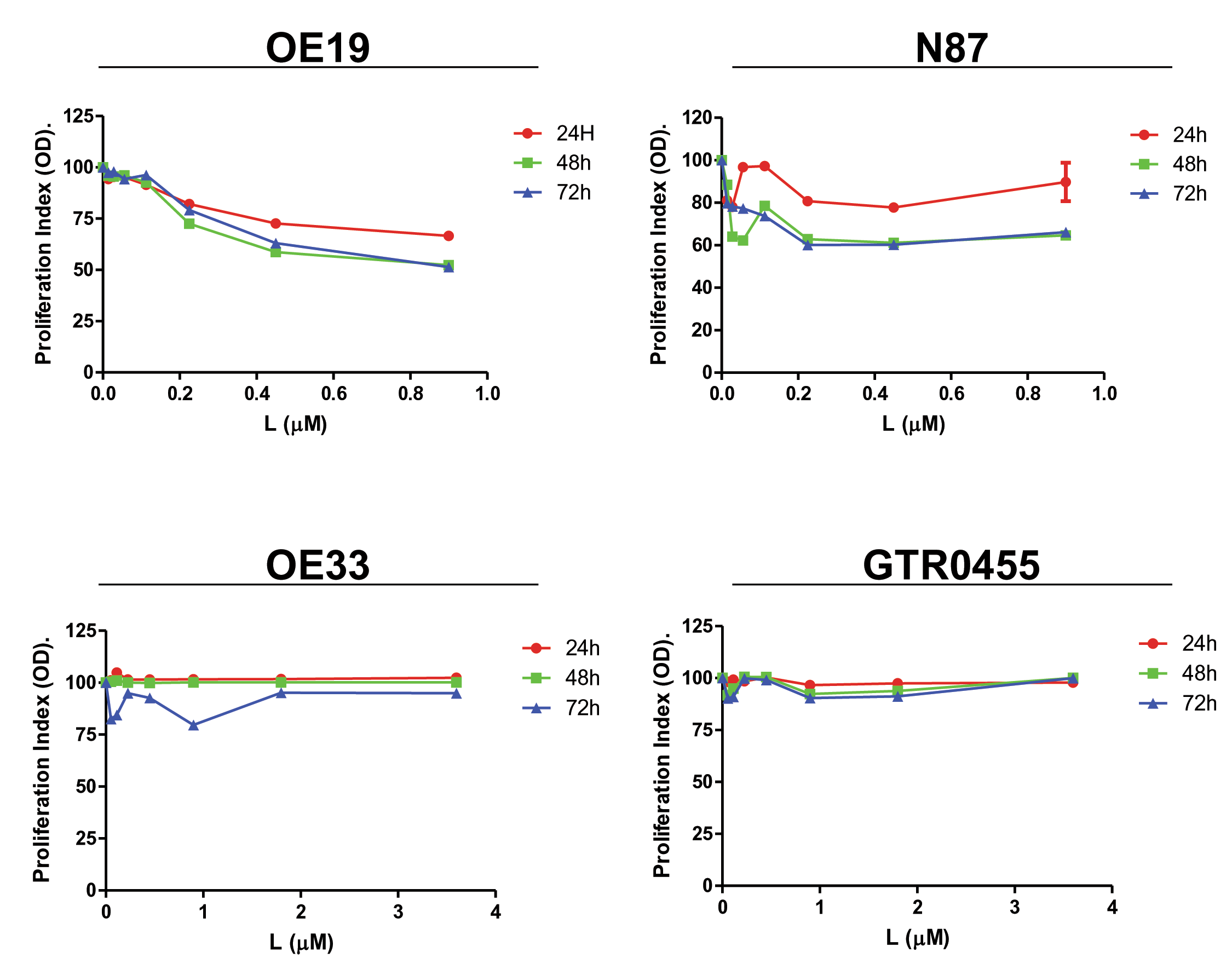

Supplement: Supplementary file 7 — Antitumor growth activity of L in OE19, N87, OE33 and GTR0455 cells cultured in adherent 2D conditions and evaluated by a WST-1-cell cytotoxicity assay. Cells were grown for 24 h, 48 h and 72 h in the presence of increasing concentrations of L. The values were normalized to the growth of the cells incubated with DMSO. (PNG 108 600 kb) [file 13402_2023_769_Fig10_ESM.png]

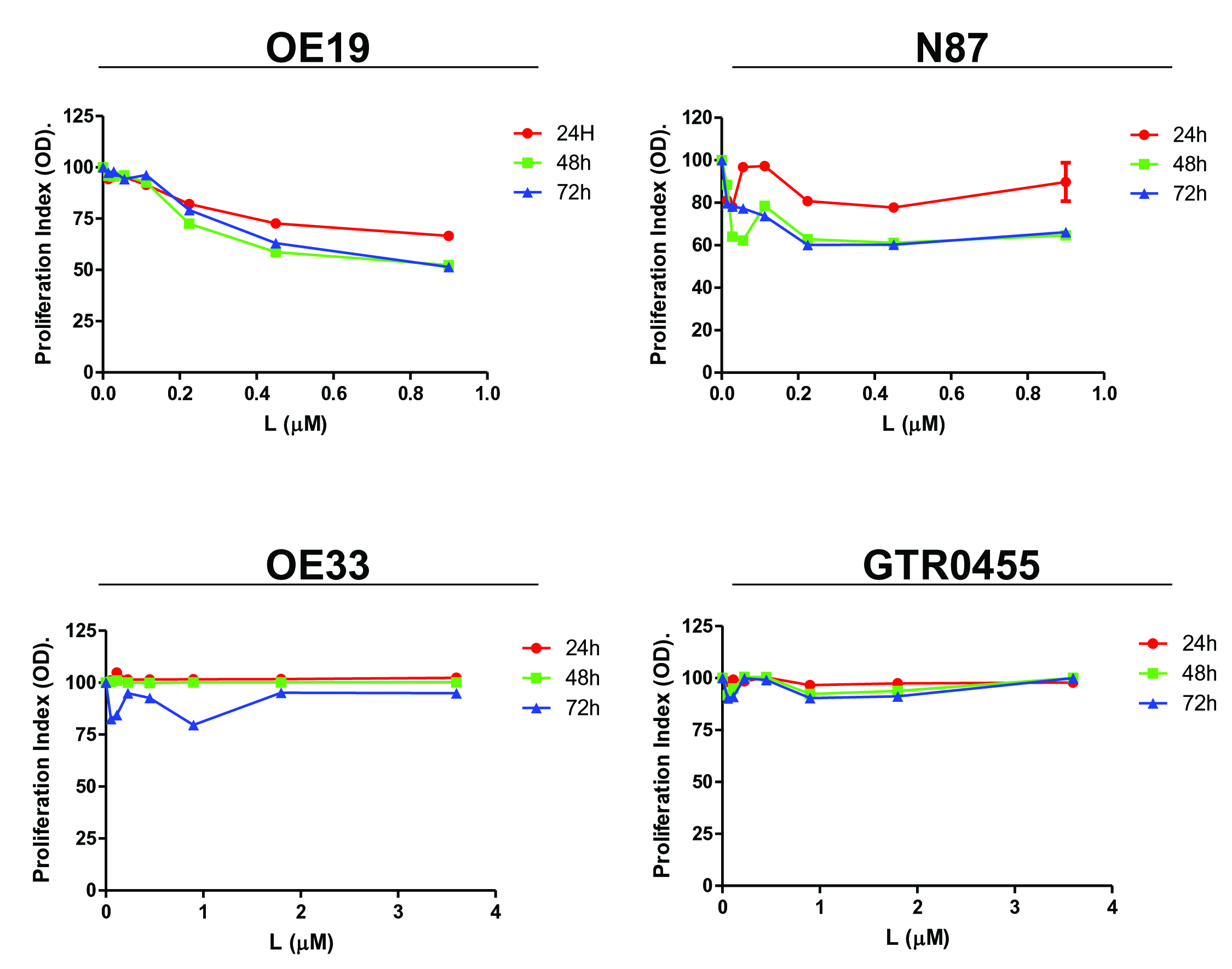

Supplement: Supplementary file 8 — Supplementary file4 (TIF 1053 KB) [file 13402_2023_769_MOESM4_ESM.tif]

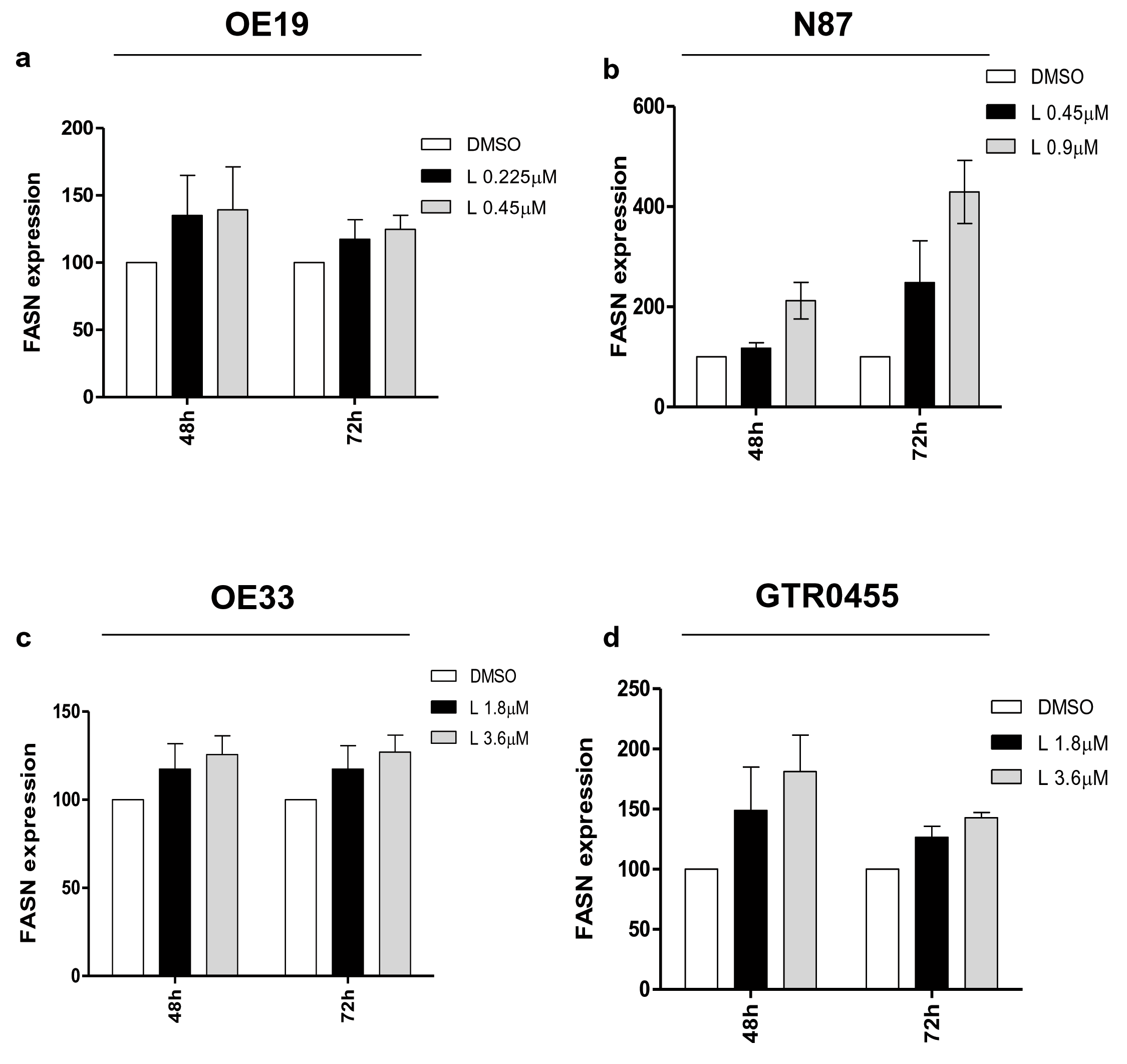

Supplement: Supplementary file 9 — Densitometric analysis of FASN expression in OE19, N87, OE33 and GTR0455 cells after treatment with lapatinib (L) or DMSO evaluated by Western Blot shown in Fig. 3a-d. Protein extracts were separated by 4-12% gradient SDS-PAGE under reducing conditions. (PNG 122 000 kb) [file 13402_2023_769_Fig11_ESM.png]

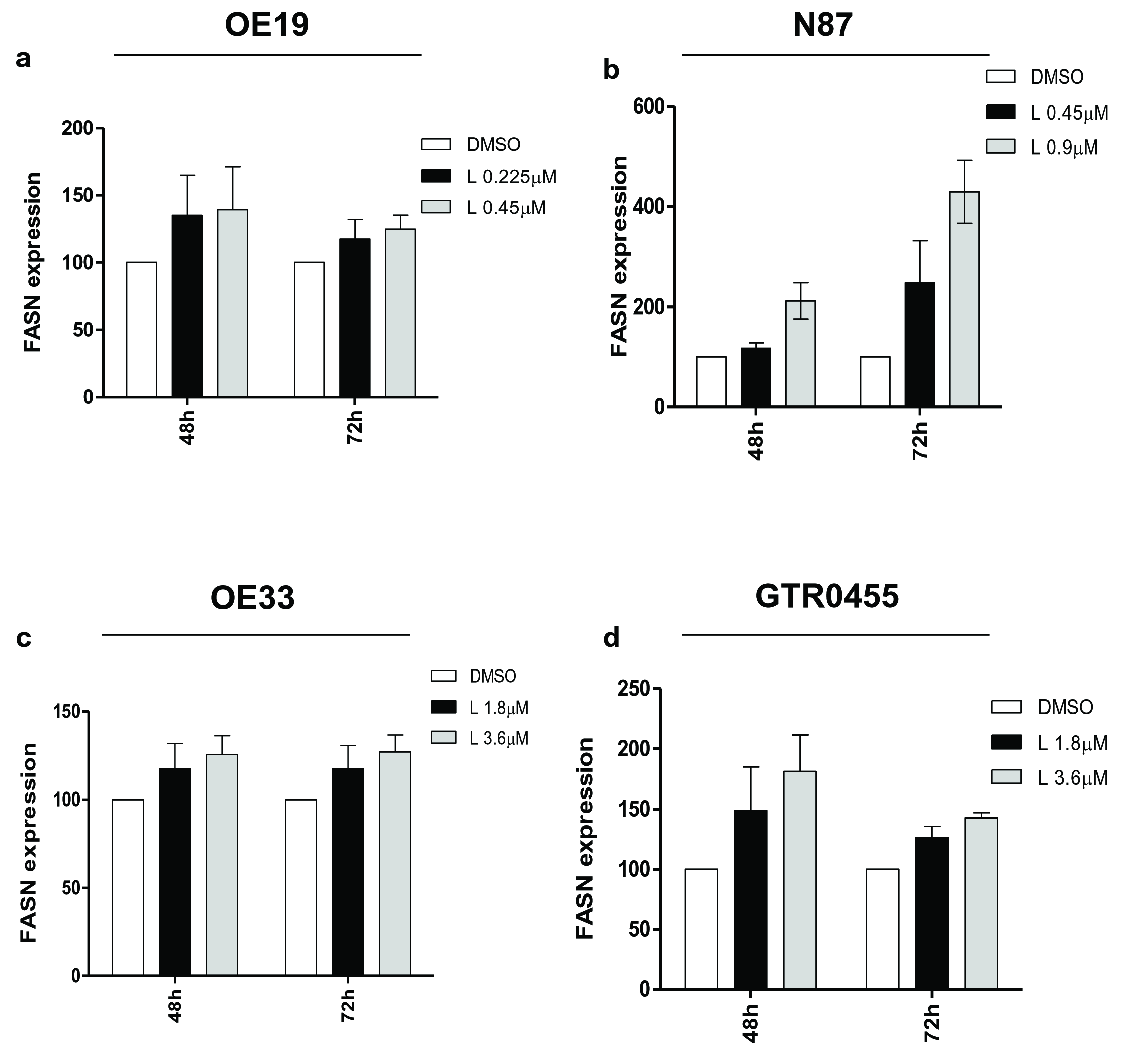

Supplement: Supplementary file 10 — Supplementary file5 (TIF 1225 KB) [file 13402_2023_769_MOESM5_ESM.tif]

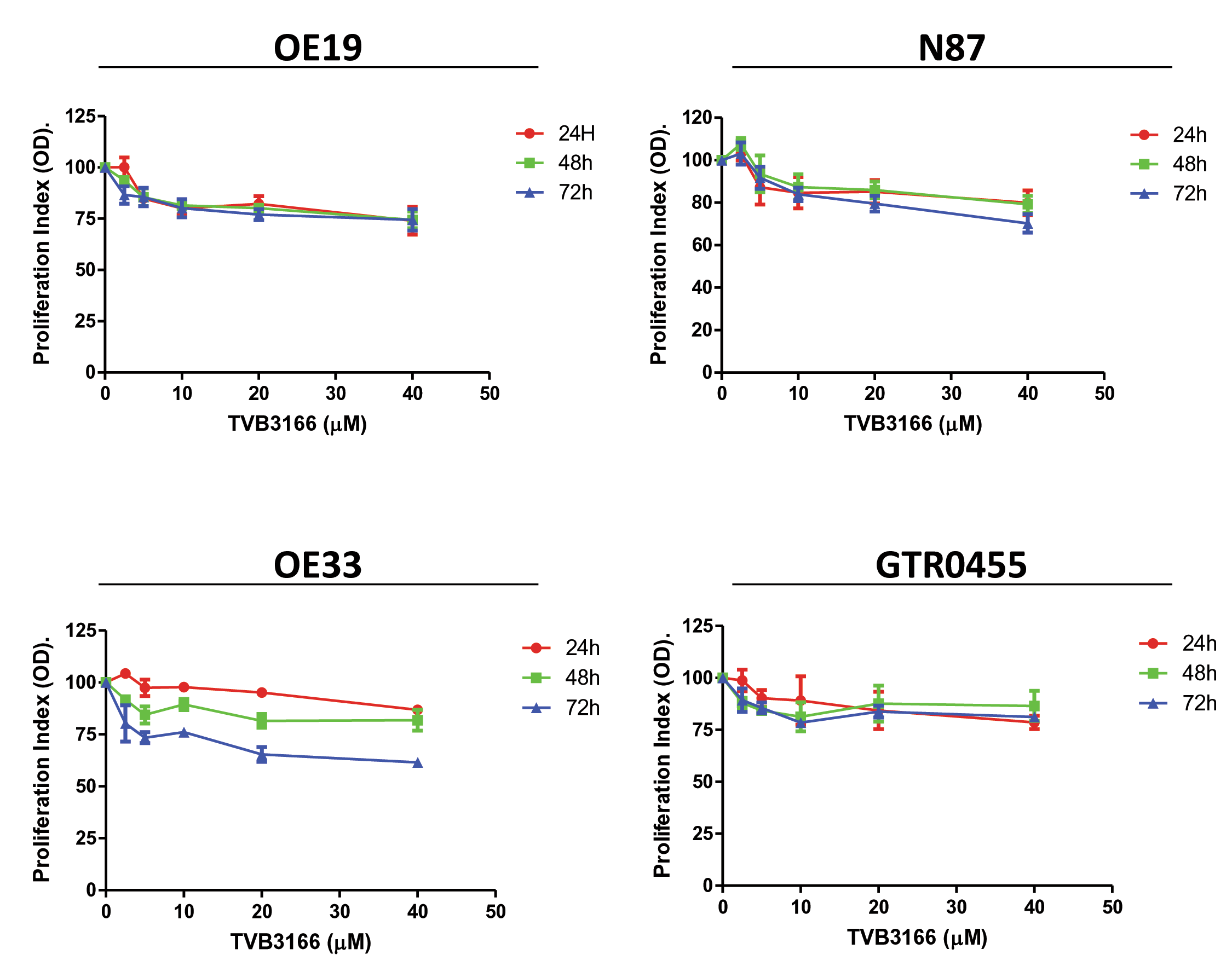

Supplement: Supplementary file 11 — Antitumor growth activity of TVB3166 in OE19, N87, OE33 and GTR0455 cells cultured in adherent 2D conditions and evaluated by a WST-1 cytotoxicity assay. Cells were grown for 24 h, 48 h and 72 h in increasing concentrations of TVB3166. The values were normalized to the growth of the cells incubated with DMSO. (PNG 155 000 kb) [file 13402_2023_769_Fig12_ESM.png]

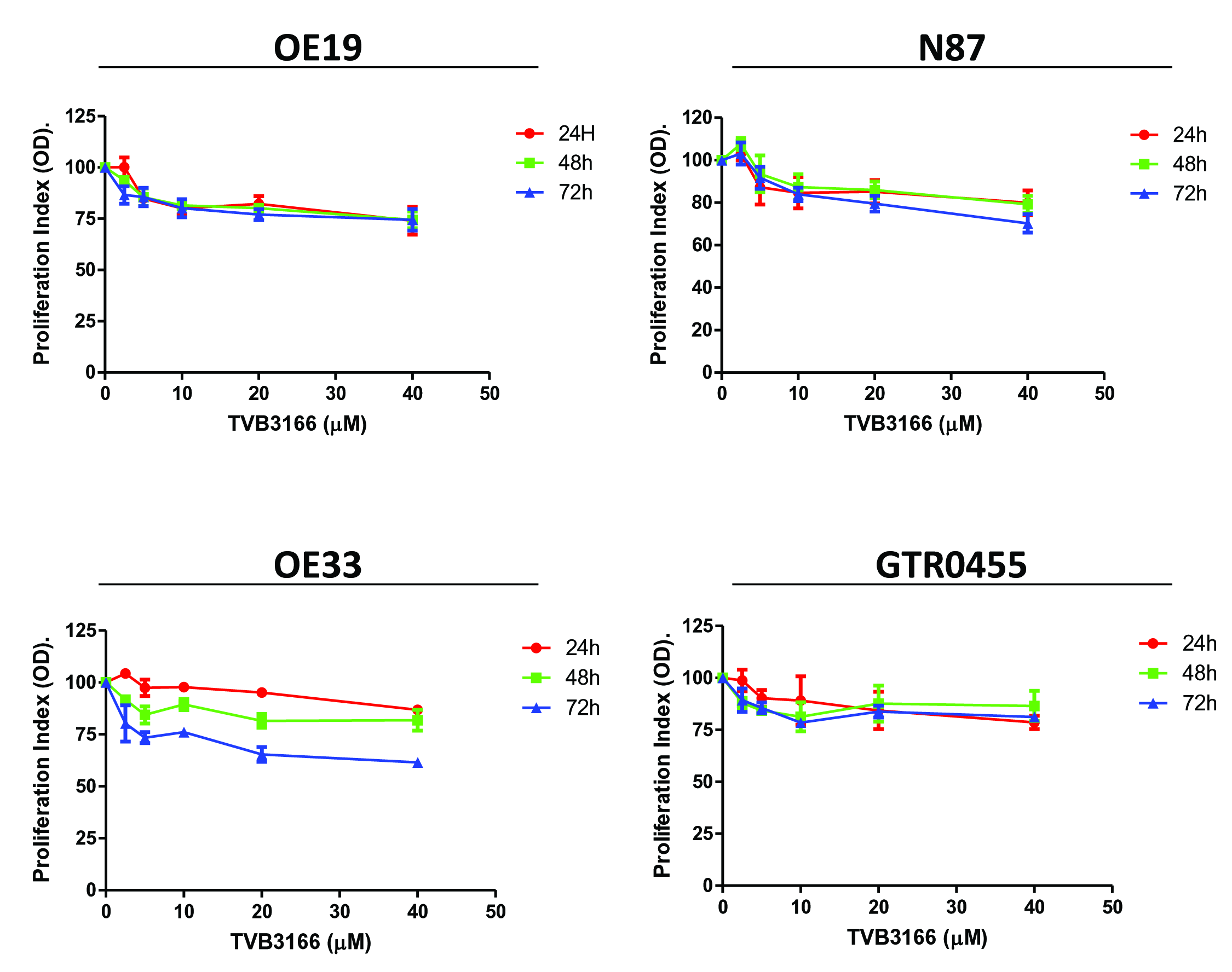

Supplement: Supplementary file 12 — Supplementary file6 (TIF 1073 KB) [file 13402_2023_769_MOESM6_ESM.tif]

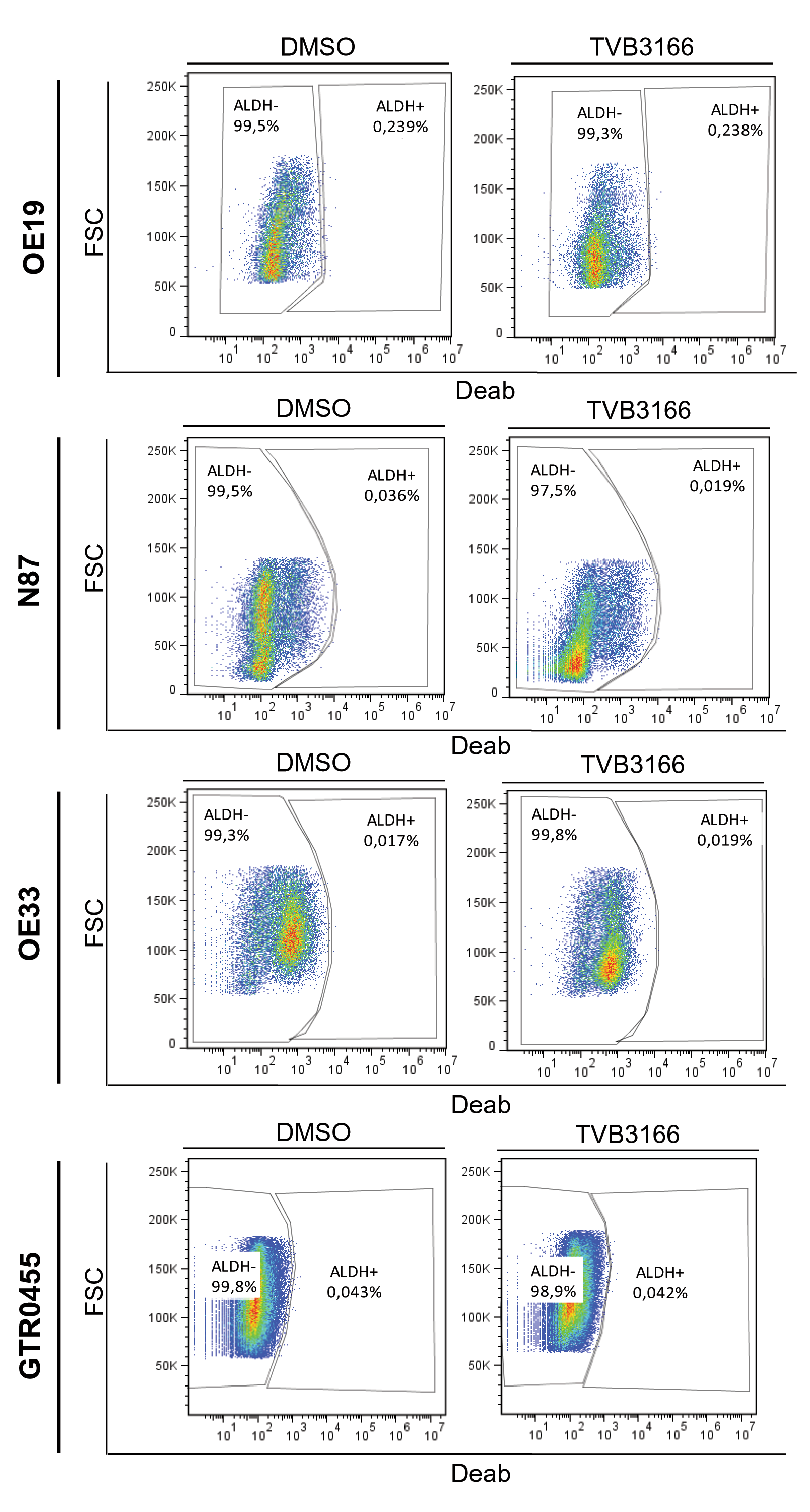

Supplement: Supplementary file 13 — Representative dot plots showing the DEAB control sample used as a background fluorescence signal for ALDEFLUOR™-stained OE19, N87, OE33 and GTR0455 cells treated with TVB3166 and DMSO. (PNG 569 000 kb) [file 13402_2023_769_Fig13_ESM.png]

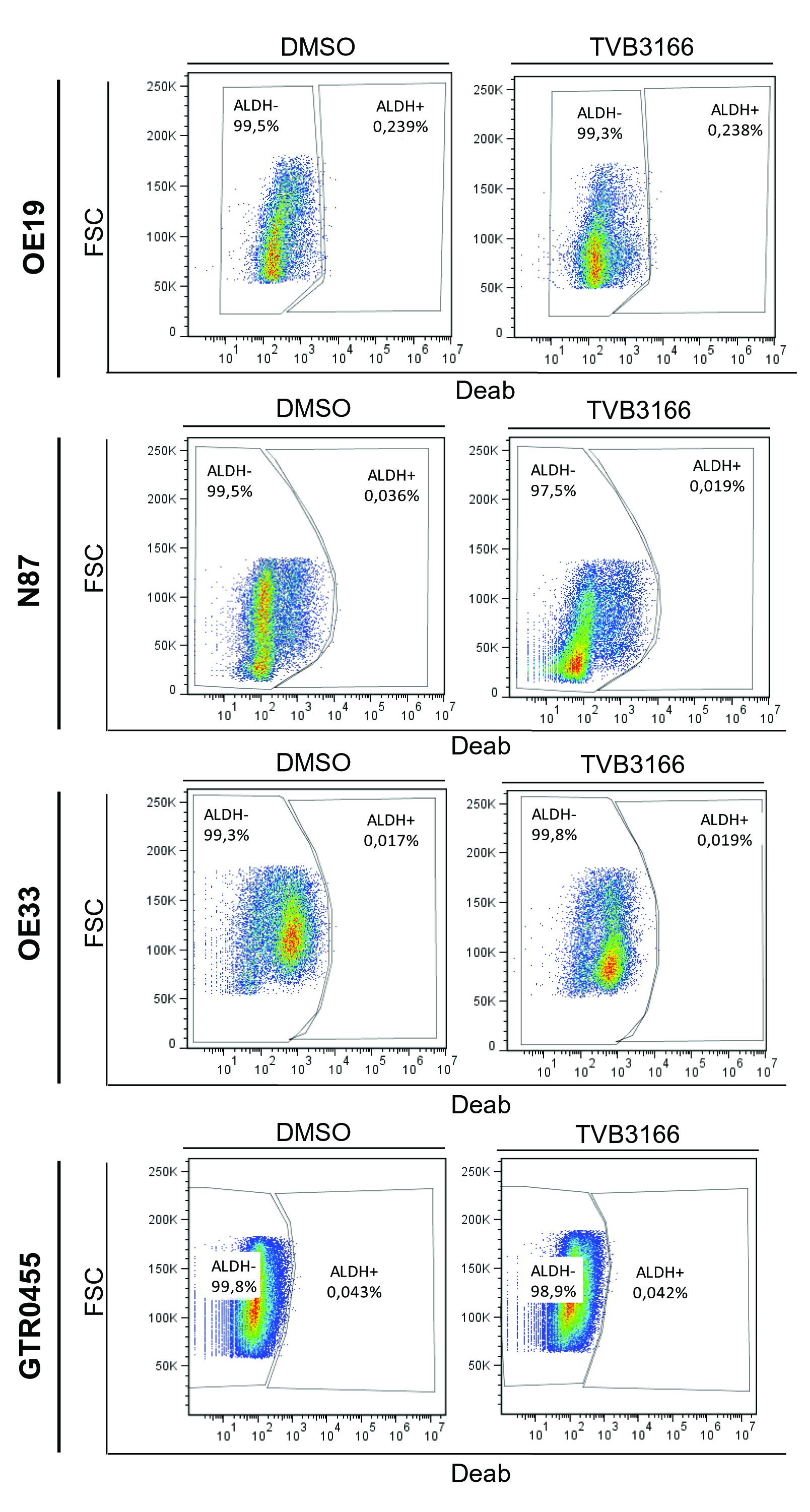

Supplement: Supplementary file 14 — Supplementary file7 (TIF 2392 KB) [file 13402_2023_769_MOESM7_ESM.tif]

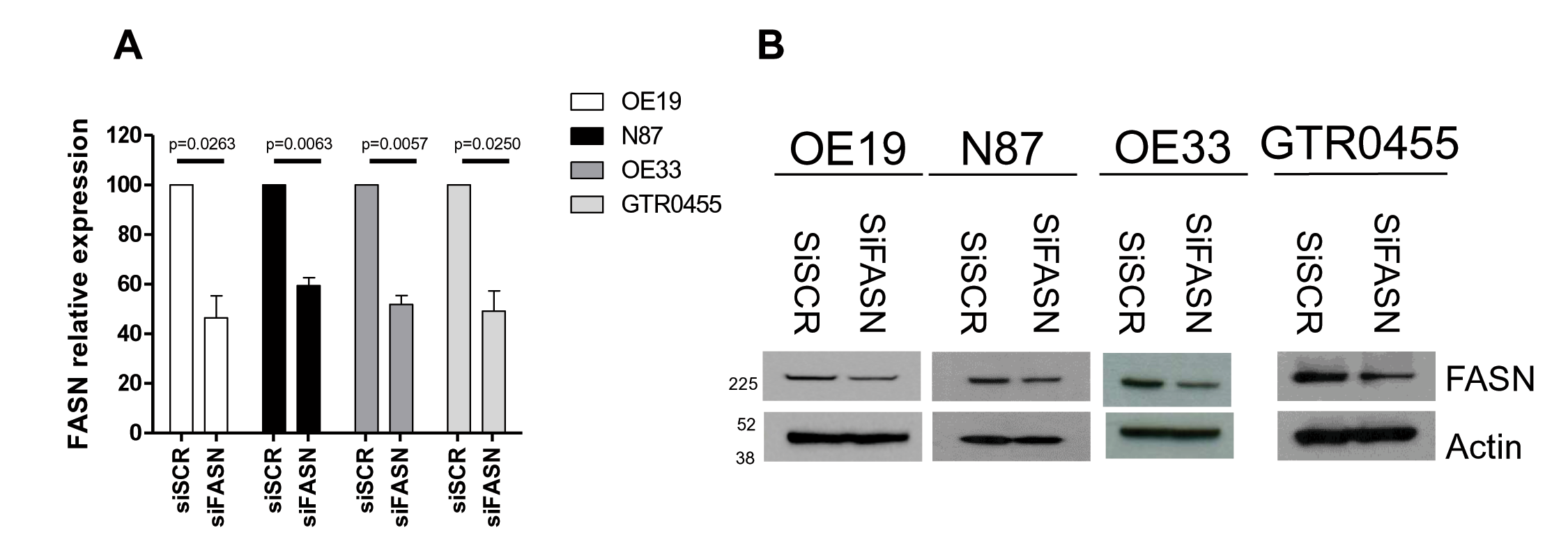

Supplement: Supplementary file 15 — a, qRT‒PCR analyses of FASN expression evaluated in OE19, N87, OE33 and GTR0455 cell lines transiently transfected with siSCR or siFASN constructs. Columns bars, means ± SEMs (n=3). Significance was calculated by a two-tailed paired t test. b, Western blot analysis of FASN expression in OE19, N87, OE33 and GTR0455 cell lines transiently transfected with siSCR or siFASN constructs. Blots were probed with FASN antibody. Actin was used to normalize protein loading. (PNG 141 000 kb) [file 13402_2023_769_Fig14_ESM.png]

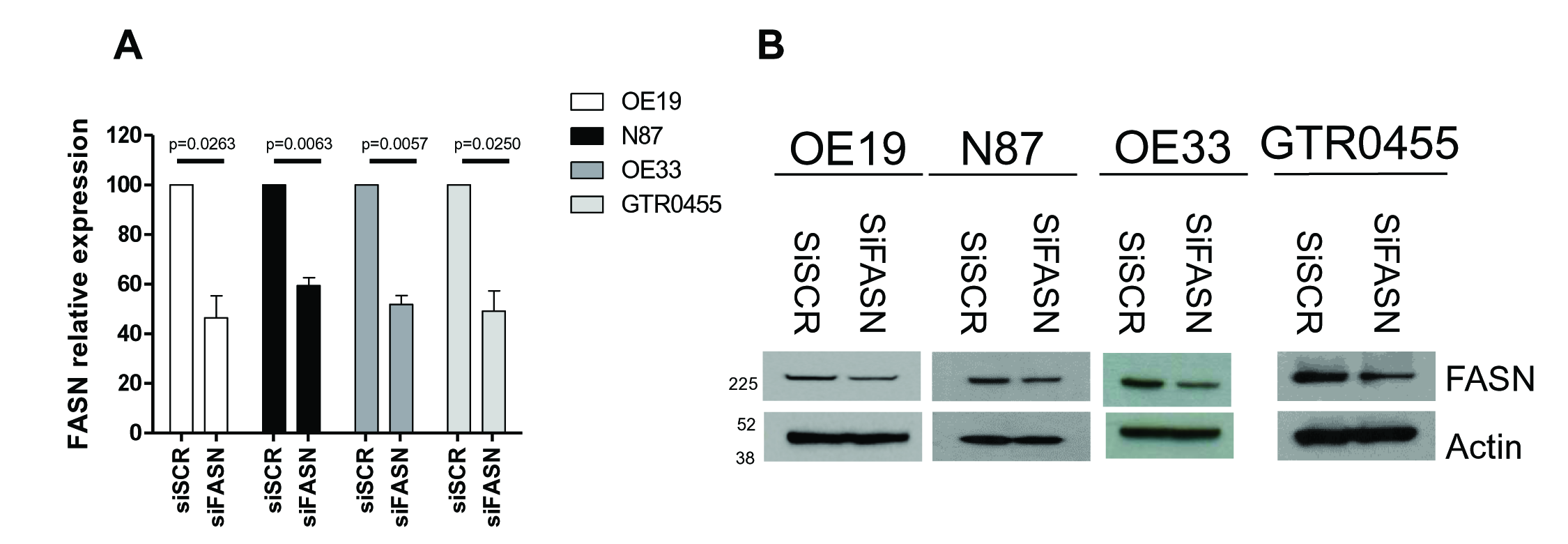

Supplement: Supplementary file 16 — Supplementary file8 (TIF 973 KB) [file 13402_2023_769_MOESM8_ESM.tif]

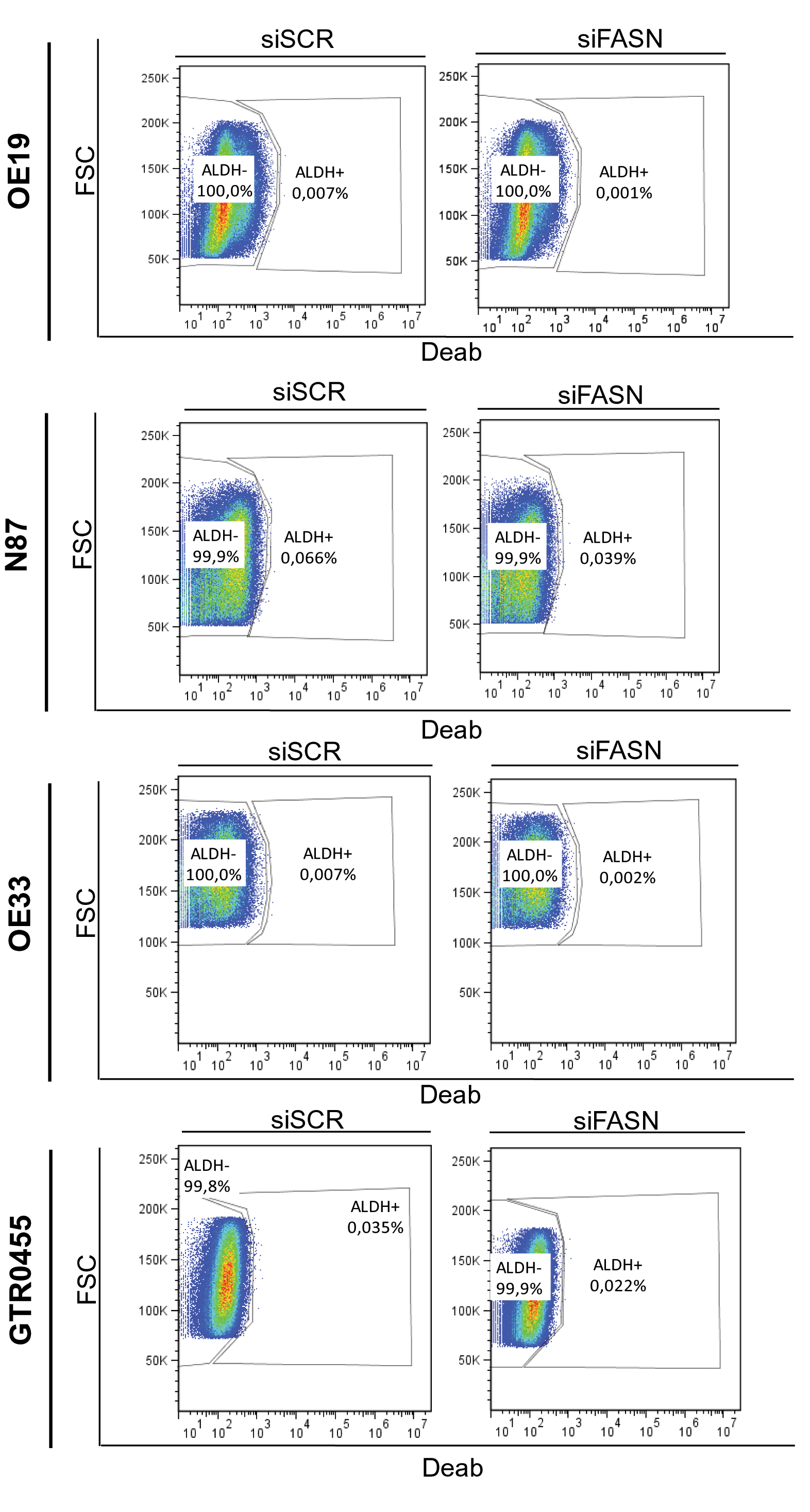

Supplement: Supplementary file 17 — Representative dot plots showing the DEAB control sample used as the background fluorescence signal for ALDEFLUOR™-stained OE19, N87, OE33 and GTR0455 cells transfected with siSCR or siFASN primers. (PNG 585 000 kb) [file 13402_2023_769_Fig15_ESM.png]

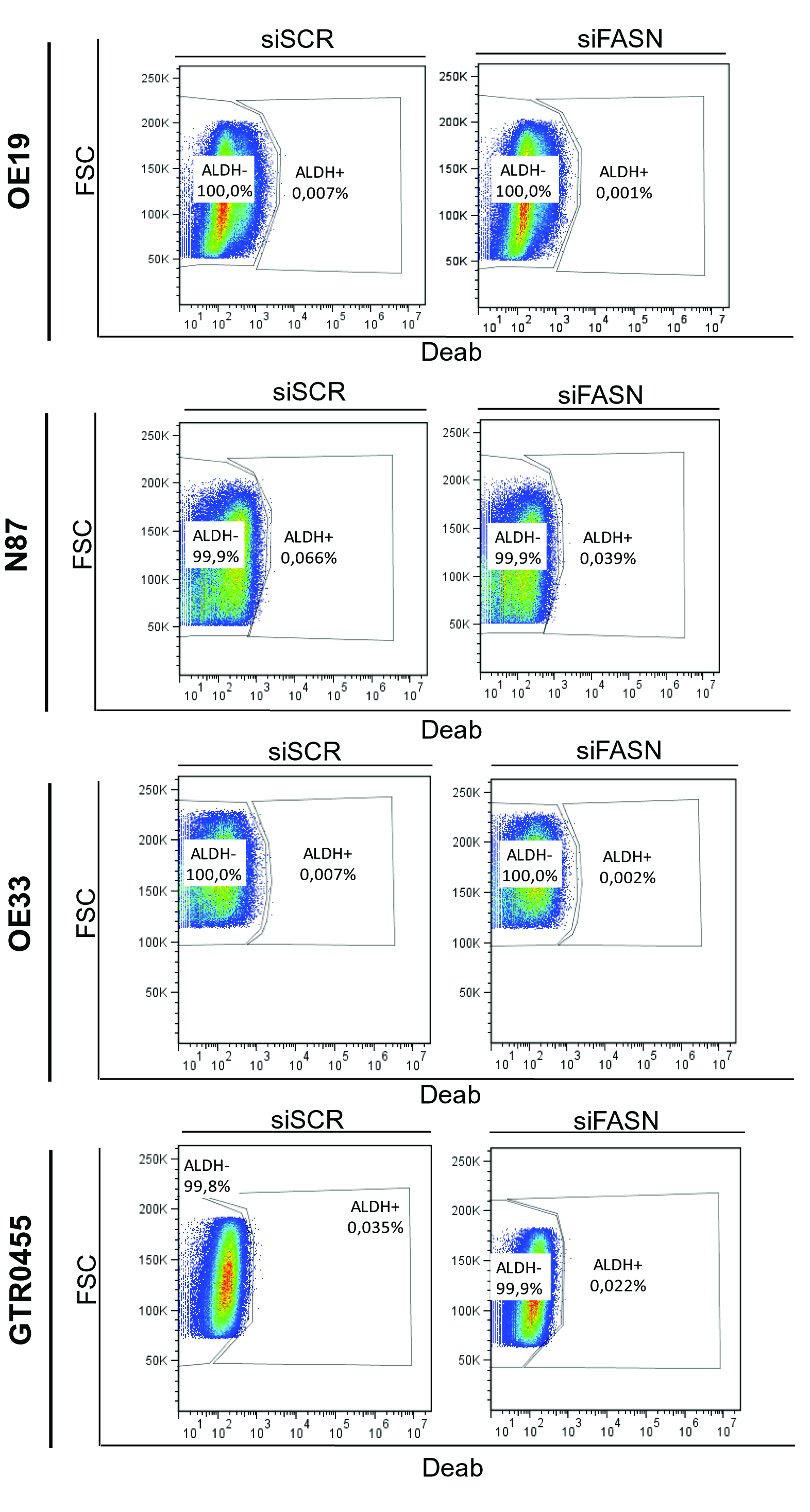

Supplement: Supplementary file 18 — Supplementary file9 (TIF 2203 KB) [file 13402_2023_769_MOESM9_ESM.tif]

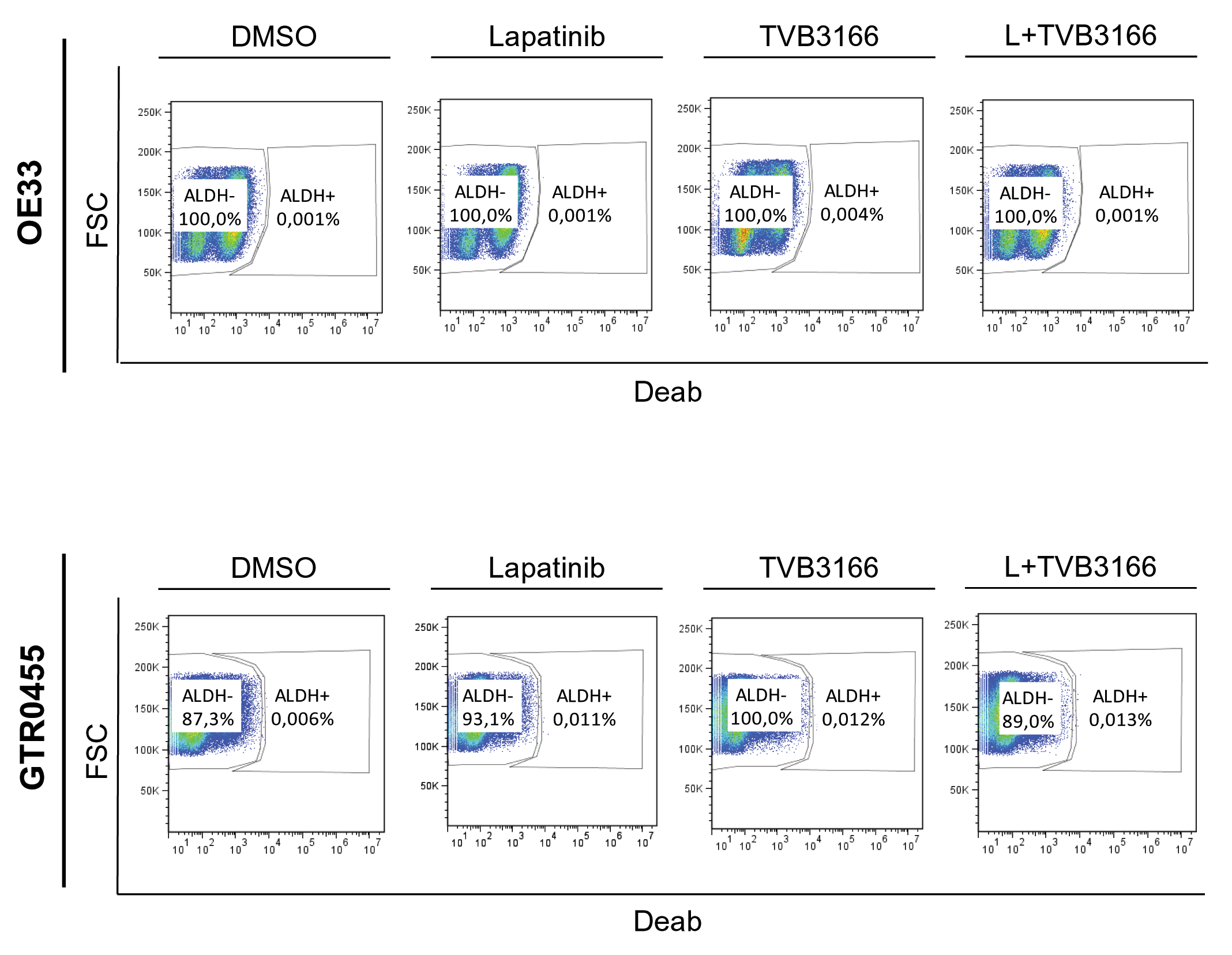

Supplement: Supplementary file 19 — Representative dot plots showing the DEAB control sample used as a background fluorescence signal for ALDEFLUOR™-stained OE33 and GTR0455 cells treated with DMSO, L, TVB3166 and L+TVB3166. (PNG 341 000 kb) [file 13402_2023_769_Fig16_ESM.png]

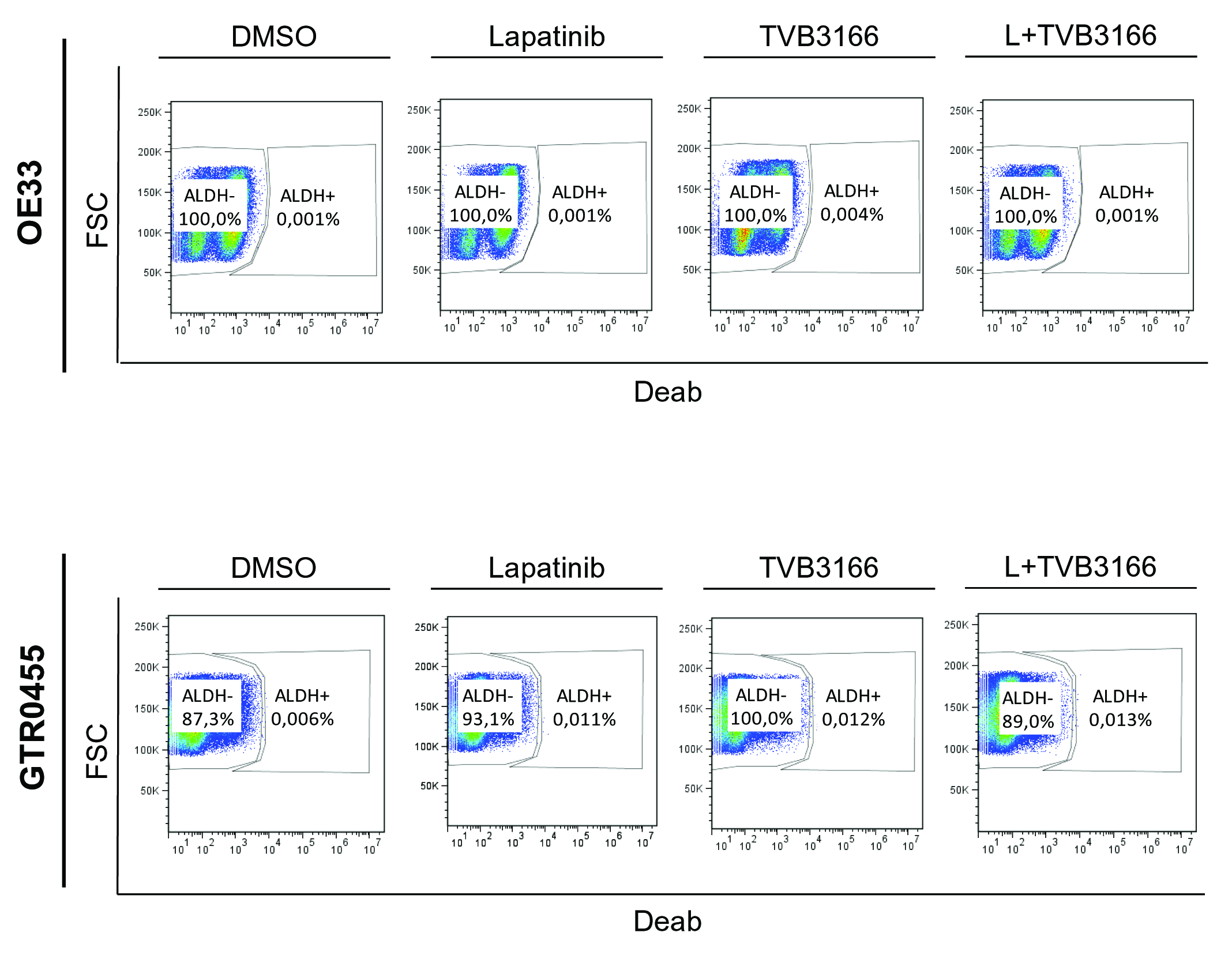

Supplement: Supplementary file 20 — Supplementary Table 1 Results of GSEA in the comparison between 3D versus 2D cell lines (TIF 1519 KB) [file 13402_2023_769_MOESM10_ESM.tif]
